# Phytochemical Diversity, Nutritional Values, and Biological Properties of *Halopithys incurva* (Hudson) Batters, 1902

**DOI:** 10.1002/fsn3.71690

**Published:** 2026-04-07

**Authors:** Youssra Aalilou, Abdelhakim Bouyahya, Kaoutar Benrahou, Mustapha Hassoun, Hanaa Moussa, Issam Ameziane El Hassani, Waleed Al Abdulmonem, Learn‐Han Lee, Gokhan Zengin, My El Abbes Faouzi

**Affiliations:** ^1^ Laboratories of Pharmacology and Toxicology, Pharmaceutical and Toxicological Analysis Research Team, Faculty of Medicine and Pharmacy Mohammed V University Rabat Morocco; ^2^ Laboratory of Human Pathologies Biology, Faculty of Sciences Mohammed V University Rabat Morocco; ^3^ Applied Phycology‐Mycology Group, Applied Botany Laboratory, Department of Biology, Faculty of Sciences Abdelmalek Essaâdi University Tetouan Morocco; ^4^ Laboratory of Medicinal Chemistry, Drug Sciences Research Center, Faculty of Medicine and Pharmacy Mohammed V University Rabat Morocco; ^5^ Department of Pathology, College of Medicine Qassim University Buraydah Saudi Arabia; ^6^ Novel Bacteria and Drug Discovery Research Group (NBDD), Microbiome Research Group, Research Centre for Life Science and Healthcare, Nottingham Ningbo China Beacons of Excellence Research and Innovation Institute (CBI) University of Nottingham Ningbo China Zhejiang China; ^7^ Microbiome and Bioresource Research Strength (MBRS), Jeffrey Cheah School of Medicine and Health Sciences Monash University Malaysia Bandar Sunway Malaysia; ^8^ Physiology and Biochemistry Laboratory, Department of Biology, Science Faculty Selcuk University Konya Turkey

**Keywords:** bioactive compounds, biological properties, *Halopithys incurva*, nutraceutical value, phytochemistry

## Abstract

*Halopithys incurva* (Hudson) Batters, 1902 is a red macroalga with a broad geographical distribution. In total, 41 chemical constituents have been characterized from *H. incurva* , including bromophenols, isoflavones, mycosporine‐like amino acids, saturated and unsaturated fatty acids, and phytohormones. In addition, this species exhibits an appreciable content of phenols, proteins, lipids, carbohydrates, and pigments, supporting its potential nutraceutical relevance. Extracts from *H. incurva* have been reported to display various biological activities, including antimicrobial, antioxidant, immunomodulatory, anti‐inflammatory, antitumoral, anti‐amyloidogenic activities, and cholinesterase‐inhibitory effects, suggesting neuroprotective potential. This review provides a comprehensive synthesis of current knowledge on the chemical composition, bioactivities, geographical distribution, taxonomy, and botanical characteristics of *H. incurva* . It also highlights critical knowledge gaps and emphasizes the need for integrated chemical, biological, and toxicological investigations, as well as in vivo validation, to better define the potential applications and limitations of this underutilized marine species.

## Graphical abstract

*Halopithys incurva* is a red macroalga distributed across the Mediterranean Sea and North‐East Atlantic, with occasional reports from the Indian Ocean. It exhibits a rich chemical diversity, including isoflavones, bromophenols, MAAs, pigments, phycobiliproteins, primary metabolites, and neuroactive compounds. It demonstrates multiple biological activities, notably antioxidant, immunomodulatory, cytotoxic, antiproliferative, anti‐amyloidogenic, neuroprotective, and broad‐spectrum antimicrobial effects.

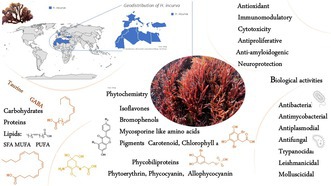

**Abbreviations**
ABTS: 2,2′‐azino‐bis (3‐ethylbenzothiazoline‐6‐sulphonic acid)
AChE: acetylcholinesterase
BChE: butyrylcholinesterase
BHA: butylated hydroxyanisole
BHT: butylated hydroxytoluene
BS: blood stage
CUPRAC: cupric reducing antioxidant capacity
DPPH: 2,2‐diphenyl‐1‐picrylhydrazyl
EC_50_
: half maximal effective concentration
FAS‐II: type II fatty acid synthesis
FRAP: ferric reducing antioxidant power
HEWL: hen egg white lysozyme
IC_50_
: inhibitory concentration required for 50% inhibition
IL‐6: Interleukin 6
iNOS: nitric oxide synthase
LC_50_
: concentration of a toxic substance lethal to half of the test animals
LS: liver stage
MAAs: mycosporine‐like amino acids
MIC: minimum inhibitory concentration
MUFA: monounsaturated fatty acids
NDs: neglected diseases
NO: nitric oxide
ORAC: oxygen radical absorbance capacity
PfFabG: beta‐ketoacyl‐ACP reductase
PfFabI: enoyl‐ACP reductase
PfFabZ: beta‐hydroxyacyl‐ACP dehydratase
PLA2: phospholipase A2
PUFA: polyunsaturated fatty acids
PVPP: polyvinylpolypyrrolidone treatment
SFA: saturated fatty acids
WHO: World Health Organization

## 1 Introduction

Over the past decade, increasing scientific attention has been directed toward marine organisms, in particular seaweeds owing to their wide range biological activities and growing relevance in biomedical and nutritional research (Mendes et al. 2022; Kumari et al. 2023; Aalilou et al. 2024; Mayer et al. 2024). Seaweeds are recognized as valuable sources of functional compounds with applications in human and animal nutrition, biotechnology, and health‐related fields, largely owing to their unique chemical composition and adaptive metabolic capabilities (Mendes et al. 2022; González‐Meza et al. 2023; Kumari et al. 2023; Dias et al. 2023). Continuous exposure to fluctuating and often harsh environmental conditions has driven seaweeds to develop specialized metabolic pathways, resulting in the biosynthesis of structurally diverse and specific secondary metabolites with notable biological activities. Phytochemically, macroalgae synthesize complex molecules that are rarely or entirely absent in terrestrial plants (Cian et al. 2015; Raúl et al. 2015; Tavares et al. 2023). Among algal groups, red macroalga (Rhodophyta) represent the most chemically diverse phylum, accounting for more than half of the bioactive algal secondary metabolites identified to date and comprising nearly 6000 species (Amminikutty 2021). Their characteristic coloration arises from specific pigment combinations carotenoids, phycobiliproteins, chlorophyll a and d, which reflecting their ecological adaptation (Leal et al. 2013; Carpena et al. 2022).

Among red macroalgae, *Halopithys incurva* (Hudson) Batters, 1902 (also historically reported as *Halopitys incurvus* ) derives its generic name from the Greek reference to “sea pine,” while the specific epithet *incurva* refers to the inwardly curved morphology of its branches. Morphologically, *H. incurva* is characterized by cylindrical, cartilaginous, tough, and shaggy dark‐red fronds reaching lengths of approximately 10–30 cm (Rodríguez‐Prieto et al. 2013; Guiry et al. 2024). It is a perennial species inhabiting rocky substrates from mid‐intertidal pools to subtidal zones. Although often described as widely distributed, *H. incurva* is not cosmopolitan, but rather exhibits a broad Mediterranean‐Atlantic and subtropical distribution (Se‐ Se‐Kwon Kim 2015; Guiry et al. 2024). Chemically, *H. incurva* is recognized as a valuable marine bioresource, containing a diverse spectrum of phytocompounds of considerable biological relevance. Reported constituents include high phenolic levels (Plaza et al. 2010; Güenaga Unzetabarrenechea 2011; Nmila and Rchid 2018; Nunes et al. 2020; Parailloux et al. 2020; Vega et al. 2020), encompassing specific classes such as mycosporine‐like amino acids (Alvarez‐Gómez et al. 2016; Parailloux et al. 2020), bromophenols (Augier and Mastagli 1956; Chantraine et al. 1973; Glombitza et al. 1974; Álvarez‐Gómez et al. 2019), and isoflavones (Klejdus et al. 2010). In addition, *H. incurva* contains appreciable amounts of lipids (Alvarez‐Gómez et al. 2016; Nunes et al. 2020; Galindo et al. 2021), proteins (Cian et al. 2015; Plaza et al. 2010; Nunes et al. 2020; Terriente Palacios et al. 2022), and carbohydrates (Plaza et al. 2010; Alvarez‐Gómez et al. 2016; Nunes et al. 2020). Moreover, it exhibits a distinctive pigment profile, including chlorophylls, carotenoids, and phycobiliproteins (Güenaga Unzetabarrenechea 2011; Cian et al. 2015; Álvarez‐Gómez et al. 2019; Vega et al. 2020; Nunes et al. 2020; Galindo et al. 2021; Thiviya et al. 2022), along with a rich mineral content (Nunes et al. 2020). Owing to this rich phytochemical profile, numerous studies aimed to explore the biological properties of *H. incurva* . Reported activities include antimicrobial effects, such as antibacterial (González del Val et al. 2001; Oumaskour et al. 2013; Khelil‐Radji et al. 2017), antiplasmodial (Spavieri et al. 2013), antifungal (Bahammou et al. 2021); Khelil‐Radji, antimycobacterial, trypanocidal, and leishmanicidal (Allmendinger et al. 2010), as well as molluscicidal effects (Patel et al. 2008). In addition, *H. incurva* exhibits pronounced antioxidant activity (Plaza et al. 2010; Güenaga Unzetabarrenechea 2011; Bouhlal et al. 2013; Zbakh et al. 2014; Alvarez‐Gómez et al. 2016; Nmila and Rchid 2018; Vasarri et al. 2020; Vega et al. 2020; Terriente Palacios et al. 2022), anti‐inflammatory and immunomodulatory properties (Abdala Díaz et al. 2011; Oumaskour et al. 2013), cytotoxic and antiproliferative effects (Allmendinger et al. 2010; Zbakh et al. 2014; Vasarri et al. 2020; Amminikutty 2021), and anti‐amyloidogenic activity (Vasarri et al. 2020). Furthermore, its potential to inhibit cholinesterase enzymes, relevant to neuroprotection, has also been reported (Kilic et al. 2021).

Based on the available literature and to the best of our knowledge, this work represents the first comprehensive synthesis review dedicated exclusively to *H. incurva* , integrating its phytochemical composition, nutraceutical value, and diverse biological activities, together with its botanical description, taxonomy, and geographical distribution. The compiled evidence highlights *H. incurva* as an underexplored marine bioresource with considerable biological potential. Nevertheless, further investigations are required to validate its pharmacological potential and to elucidate the underlying molecular mechanisms of action. Given the limited number of studies focused on the detailed chemical characterization of *H. incurva* , future research should prioritize metabolite identification and structure–activity relationships, while considering the influence of extraction solvent, methodology, seasonal variation, and geographical origin. Additionally, exploration of its potential effects on other biological pathways including diabetes, viral infections, hepatoprotection, cardiovascular and renal health, neurocognitive function, wound healing, and cosmetic applications remains a promising direction for future research. In addition, comprehensive investigations of the toxicological and nutraceutical profiles of *H. incurva* are necessary, especially considering that dietary supplementation with this macroalga has been shown to exert pronounced positive effects on immune responses and antioxidant status (Hoseinifar et al. 2022).

## 2 Research Methodology

A comprehensive literature survey was conducted to collect all relevant information pertaining to *H. incurva*. Data were retrieved from scientific research databases, including Algae Database (https://www.algaebase.org/), as well as electronic resources such as Google Scholar, PubMed, Wiley Online, Web of Science, Springer Link, and ScienceDirect. The search strategy was guided by the use of specific keywords, including “ *H. incurva* ,” “*Halopithys incurva*,” “*Halopithys incurvu*,” “red macroalgae phytochemistry,” “red seaweed activities,” “red algae properties,” “ *H. incurva* activities,” “ *H. incurva* and effect,” “geographical distribution of *H. inurva*,” “the chemical composition of *H. incurva* ,” “Phytochemical profile of *H. incurva* .” An initial broad database search yielded a large number of records. After refinement, relevance screening, and removal of duplicates, 118 studies were retained for inclusion in this review. Chemical structures were drawn using Chem‐Drawn software, while compound classifications and structural validation were performed using the PubChem database (https://pubchem.ncbi.nlm.nih.gov/). Reference management, data organization, and manuscript drafting were carried out using Zotero software.

## 3 Results and Discussion

### 3.1 General Description

**FIGURE 1:**
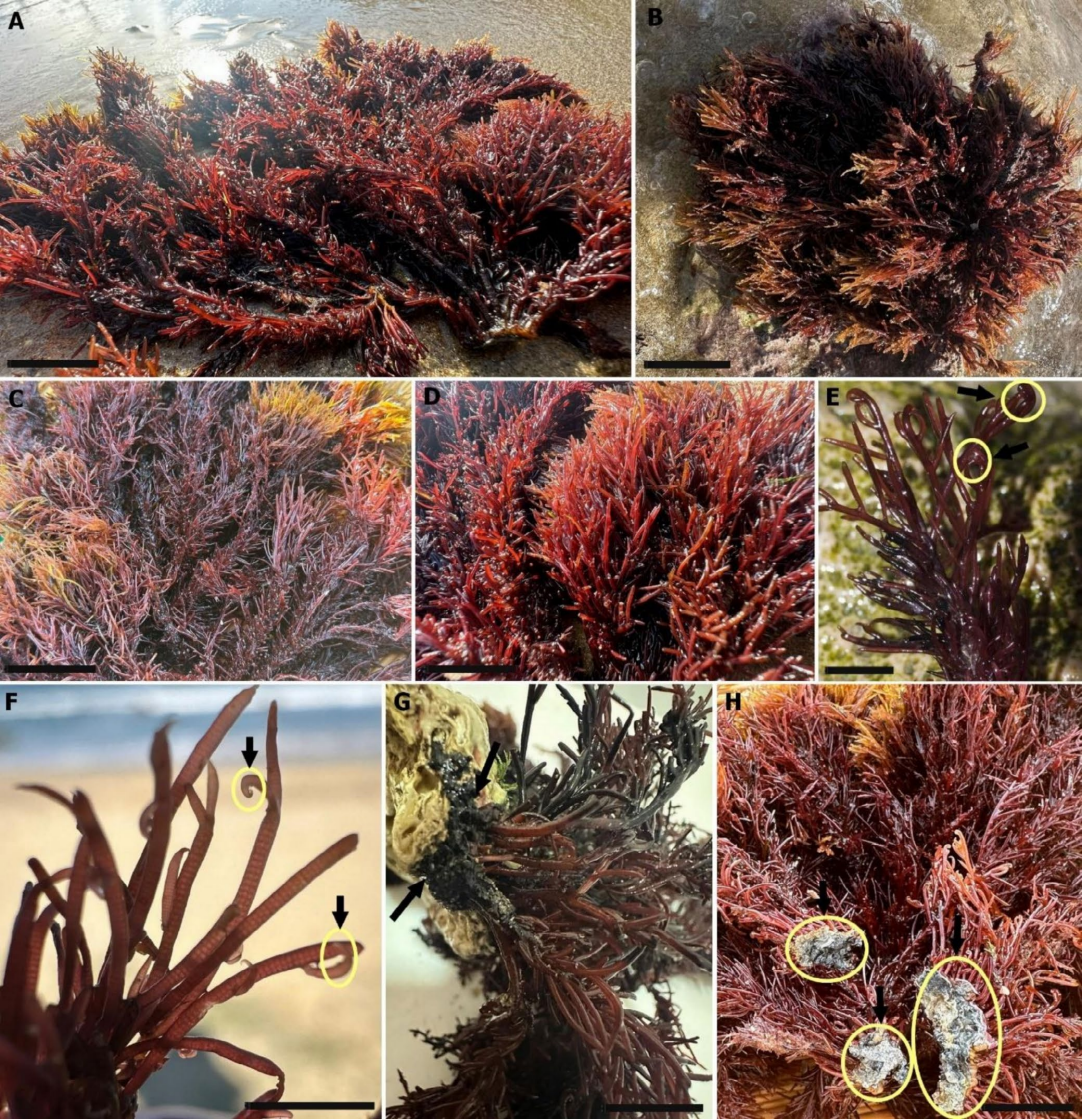
*Halopithys incurva* (Hudson) Batters. (A, B) General habit; (C, D) densely branched erect axes; (E, F) details of branches, showing laterals with inrolled apices (arrows); (G, H) fixation of axes to substratum by a solid disciform holdfast (arrows). Scale bars: A, B: 2 cm; C: 3 cm; D–H: 1 cm.

*Halopithys incurva* is a tufted plant, 10 to 30 cm in height, brownish red to dark red in color. The thalli are dorsiventrally organized and consist of dense tufts of terete axes with a cartilaginous consistency and appearance (Figure 1A,B). The erect axes are densely branched, with a flat‐topped, obconical to irregular outline (Figure 1C,D). Main axes bear an irregular arrangement of lateral branches and densely covered below with short lateral branchlets. The branches typically feature a double row of short, pointed ramuli on the upper side, with the ramuli being straight, curved, or hooked and slightly tapered at the base (Figure 1E,F). Branches of the main axes are alternate or sometimes subdichomous, simple or pectinate in the lower parts, and commonly branched in upper parts. They are often curved and hooked. The axes are attached to substratum by a solid disciform holdfast, up to 2 cm in diameter (Figure 1G,H).

### 3.2 Taxonomy

*Halopithys incurva* (Hudson) Batters, 1902 is classified in the following hierarchical classification order (Guiry et al. 2024; MNHN and OFB 2024). Kingdom: Plantae Haeckel, Subkingdom: Biliphyta, Infrakingdom: Rhodaria Wettstein, Phylum: Rhodophyta, Subphylum: Eurhodophytina Saunders and Hommers, Class: Florideophyceae Cronquist, Subclass: *Rhodymeniophycidae*, Order: Ceramiales Oltmanns, Family: Rhodomelaceae Aresch., Tribe: Amansieae Schmitz, Genus: *Halopithys* Kütz., and Species: *Halopithys incurva* (Hudson) Batters 1902.

### 3.3 Biogeographical Distribution of *H. incurva*

*Halopithys incurva* is a perennial red macroalga occurring from mid‐intertidal rock pools to the subtidal zone, where it is typically attached to rocky substrates (Amminikutty 2021; Guiry et al. 2024). Based on available checklists and floristic records, *H. incurva* exhibits a broad but regionally structured geographical distribution, mainly across warm‐temperate to subtropical marine regions. Its reported distribution is summarized in Table 1.

**TABLE 1:** Geographical distribution of *Halopithys incurva* reported in literature.

Region	Country/area	References
Africa	Morocco	(Oumaskour et al. 2013; Bouhlal et al. 2013; Zbakh et al. 2014; Moussa et al. 2018; Nmila and Rchid 2018; Bahammou et al. 2021)
	Mauritania	(Michael John et al. 2004)
	Algeria	(Garreta et al. 2001; Khelil‐Radji et al. 2017)
	Tunisia	(Garreta et al. 2001)
	Libya	
Middle East	Cyprus	(Taskin et al. 2013; Tsiamis et al. 2014)
	Egypt	(Shabaka 2018)
	Lebanon	(Lakkis 2003)
	Turkey	(Kilic et al. 2021; Oğuz and Sevilay 2022)
South‐West Asia	Sri Lanka	(Silva et al. 1996; Boudouresque et al. 2022).
Europe (Mediterranean and NE Atlantic)	Adriatic Sea	(Garreta et al. 2001)
	Apulia (Italy)	(Bottalico et al. 2016)
	Balearic Islands (Spain)	(Garreta et al. 2001; Joher et al. 2012)
	Corsica	(Marta and Enric 2010)
	France	(Boudouresque et al. 2022; Burel et al. 2019)
	Greece	(Sartoni and de Biasi 1999; Tsiamis et al. 2014)
	Netherlands	(Stegenga et al. 1997)
	Sardinia (Italy)	(Garreta et al. 2001)
	Italy	(Vasarri et al. 2020)
	Spain	(Alvarez‐Gómez et al. 2016)
Northern Atlantic Europe	Britain and Ireland	(Allmendinger et al. 2010)
Macaronesia (North Atlantic Ocean)	Madeira	(Nunes et al. 2020)
	Canary Islands	(Afonso‐Carrillo 2014)

### 3.4 Phytochemistry

*Halopithys incurva* has been reported to contain a diverse range of bioactive compounds, highlighting its potential for nutraceutical and functional food applications. All identified compounds are listed in Table 2.

**TABLE 2:** Identified compounds and substances reported in *Halopithys incurva*.

No	Class	Molecular formula	Compound	References
1	Bromophenols	C_6_ (SO_3_K)_2_ Br (OH)_2_ COOH	Unknown	(Augier and Mastagli 1956)
2		C_8_H_6_Br_2_O_3_	3,5‐dibromo‐4hydroxyphenylacetic acid	(Chantraine et al. 1973)
3		C_9_H_6_Br_2_O_4_	3,5‐dibromo‐4 hydroxyphenylpyruvic acid	
4		C_7_H_7_BrO_3_	3‐Brom‐4, 5‐dihydroxybenzylalcohol	(Glombitza et al. 1974)
5		C_7_H_7_BrO_2_	3‐Brom‐4‐hydroxybenzylalcohol	
6		C_8_H_9_BrO_6_S	3‐bromo‐4,5‐dihydroxyphenylethanol sulfate	(Álvarez‐Gómez et al. 2019)
7	Isoflavones	C_15_H_10_O_5_	Genistein	(Klejdus et al. 2010)
8		C_16_H_12_O_4_	Formononetin (Biochanin B)	
9		C_16_H_12_O_5_	Biochanin A	
10		C_21_H_20_O_9_	Daidzin	
11		C_21_H_20_O_10_	Genistin	
12		C_22_H_22_O_9_	Ononin	
13		C_22_H_22_O_10_	Sissotrin	
14		C_15_H_10_O_4_	Daidzein	
15	MAAs	C_10_H_16_N_2_O_5_	Palythine	(Alvarez‐Gómez et al. 2016; Parailloux et al. 2020)
16		C_10_H_15_NO_6_	Mycosporine‐glycine	(Parailloux et al. 2020)
17		C_12_H_20_O_6_N_2_	Asterina‐330	
18		C_13_H_22_O_8_N_2_S	Unknown	
19		C_13_H_20_N_2_O_5_	Palythene/Usujirene	
20		C_13_H_22_N_2_O_6_	Palythinol	(Alvarez‐Gómez et al. 2016; Parailloux et al. 2020)
21		C_13_H_20_N_2_O_8_	Shinorine	
22		C_14_H_22_N_2_O_8_	Porphyra‐334	(Alvarez‐Gómez et al. 2016)
23	SFAs	C_14_H_28_O_2_	Myristic acid	(Galindo et al. 2021)
24		C_15_H_30_O_2_	Pentadecanoic acid	
25		C_16_H_32_O_2_	Palmitic acid	
26		C_17_H_34_O_2_	Margaric acid	
27		C_18_H_36_O_2_	Stearic acid	
28	MUFAs	C_16_H_30_O_2_	Palmitoleic acid	
29		C_18_H_34_O_2_	Oleic acid	
30	ω‐3‐PUFAs	C_18_H_30_O_2_	Linolenic acid	
31		C_18_H_28_O_2_	Stearidonic acid	
32		C_20_H_30_O_2_	Eicosapentaenoic acid	
33		C_22_H_32_O_2_	Docosahexaenoic acid	
34	ω‐6‐ PUFAs	C_18_H_32_O_2_	Linoleic acid	
35		C_20_H_34_O_2_	Dihomo‐gamma‐linolenic acid	
36		C_20_H_32_O_2_	Arachidonic acid	
37	PGRs	C_10_H_9_NO_2_	Indole‐3‐acetic acid	(Yalçın et al. 2023)
38		C_10_H_9_N_5_O	Kinetin	
39		C_10_H_13_N_5_O	Zeatin	
40		C_12_H_11_N_5_	6‐benzyl amino purine	
41		C_15_H_20_O_4_	Abscisic acid	

Abbreviations: MAAs, mycosporine‐like amino acids; MUFAs, monounsaturated fatty acids; PGRs, plant growth regulators; PUFAs, polyunsaturated fatty acids; SFAs, saturated fatty acids.

#### 3.4.1 Phenolic Profile

Phenolic compounds are widely abundant in macroalgae, with more than 8000 structures identified to date (Sadeghi et al. 2024). In marine algae, these compounds are primarily recognized for their antioxidant properties, arising from their aromatic hydroxylated structures that enable free‐radical scavenging and metal chelation (Jimenez‐Lopez et al. 2021; Lomartire and Gonçalves 2023; Tavares et al. 2023). Algal phenolics encompass several classes including flavonoids, bromophenols, phlorotannins, coumarins, lignins, and lignans (Goksen 2023; Jimenez‐Lopez et al. 2021; Leão et al. 2023; Lomartire and Gonçalves 2023; Tavares et al. 2023; Zhao et al. 2023), and are often synthesized in response to the environmental stressors (Jimenez‐Lopez et al. 2021; Lomartire and Gonçalves 2023). Red macroalgae are generally reported to contain higher flavonoids levels than green and brown algae (Yoshie‐Stark et al. 2002; Jimenez‐Lopez et al. 2021), while both red and green algae are notable sources of bromophenols and phenolic terpenoids (Sadeghi et al. 2024).

*H. incurva* has been widely investigated for its phenolic contents and is frequently identified as a phenolic‐rich red seaweed. Nmila and Rchid (2018) reported a total phenolic content (TPC) of 112 ± 0.62 mg EAG/g of dry extract in crude extract. However, after delipidation the chloroform extract exhibited the highest TPC, reaching 303 ± 4.21 mg EAG/g of dry extract, whereas the isopropanolic extract showed markedly lower levels of 57 ± 0.64 mg EAG/g of dry extract. These results indicate that a substantial proportion of phenolic compounds in *H. incurva* are of moderate polarity and are more efficiently recovered in chloroform after lipid removal, highlighting the influence of solvent polarity on phenolic extraction efficiency.

Furthermore, the influence of cultivation and pretreatment on TPC has been also documented. Vega et al. (2020) reported that wild‐collected sample of *H. incurva* exhibited higher TPC (4.86% ± 0.02% DW) than cultivated samples (3.54% ± 0.02% DW), indicating that aquaculture conditions may reduce phenolic accumulation. Consistently, across all screened seaweeds, the authors observed lower TPC values in cultivated specimens. Similar trends were reported by Güenaga Unzetabarrenechea (2011), who demonstrated that both cultivation and polyvinylpolypyrrolidone (PVPP) pretreatment significantly reduced TPC levels, suggesting that extraction methodology and cultivation strongly affect phenolic levels. In that study, extracts from harvested *H. incurva* exhibited a TPC of 4.86% ± 0.00% DW, which decreased to 1.46% ± 0.00% DW following PVPP treatment. Likewise, cultivated samples showed lower TPC of 3.54% ± 0.02% DW, with a further reduction to 0.91% ± 0.02% DW after PVPP treatment. Notably, *H. incurva* exhibited the highest TPC among all algae tested when quantified using the Folin–Ciocalteu method. Following PVPP treatment, no detectable TPC was observed in green or red algae, except *H. incurva* , which exhibited an approximate 70% reduction. In contrast, the brown algae *Fucus spiralis* and *Cystoseira abies‐marina* retained measurable TPC levels after PVPP treatment, with decreases of 20%–30%. The reduction in total phenolic content following PVPP treatment reflects the non‐specific nature of the Folin–Ciocalteu assay and underscores the need for complementary chemical analyses to accurately quantify phenolic compounds.

Extraction temperature was shown to significantly affect TPC of *H. incurva* when subcritical water extraction was applied. Plaza et al. (2010) reported that increasing the extraction temperatures from 100°C to 200°C enhanced phenolic recovery, with TPC of 33.04 ± 3.43 and 41.78 ± 8.15 mg gallic acid/g extract, respectively. Across all screened species, raising the extraction temperature to 200°C resulted in increased TPC values. At 100°C, *H. incurva* exhibited a higher TPC than *Sargassum vulgare* , *Sargassum muticum* , *Porphyra* spp., *Undaria pinnatifida*, and *Cystoseira abies‐marina*, and also demonstrated the most pronounced antioxidant properties. However, compared with these algae, *H. incurva* did not display a marked increase in TPC when the extraction temperature was elevated to 200°C. Although this observation was not explicitly discussed by the authors, the effect of temperature on phenolic recovery is generally attributed to enhanced cell wall disruption, improved solubility, and the release of bound phenolic compounds. Nevertheless, the modest temperature‐dependent increase observed for *H. incurva* suggests that most of its phenolic compounds are readily extractable at lower temperatures, with relatively few tightly bound phenolic fractions requiring harsher extraction conditions.

Geographic origin also influenced TPC, as samples collected from different coastal sites exhibited measurable variability (Nunes et al. 2020). In that study, *H. incurva* sample collected from Abas do Rio (AR) and Calhau da Serra de For (CSF) showed TPC levels of 16.52 ± 0.76 and 14.99 ± 0.19 mg GAE.g^−1^dw, respectively. Among the 19 seaweeds analyzed, *H. incurva* ranked fourth in TPC. Notably, *H. incurva* exhibited a higher TPC than several brown algae, including *Cystoseira compressa* , *Dictyota dichotoma* , *Halopteris filicina* , *Halopteris scoparia* , *Lobophora variegata* , and *Sargassum vulgare* . Meanwhile, no detectable TPC was observed in the red algae *Asparagopsis taxiformis* and *Corallina officinalis* , the green algae *Ulva intestinalis* and *Ulva* sp., and the brown alga *Padina pavonica*. The difference in total phenolic content between the two sampling sites was pronounced and likely reflects site‐specific environmental influences that modulate phenolic biosynthesis in macroalgae, such as light intensity, temperature, salinity, hydrodynamic conditions, biotic pressure, and nutrient availability. However, detailed information on habitat characteristics or explicit justification for the selection of the two stations was not provided, and formal statistical significance of the observed differences was not reported. In another survey, Parailloux et al. (2020) investigated the impact of seasonality in the TPC of hydroalcoholic extract (HIE) of *H. incurva* harvested in May (HIE/m) and November (HIE/n). The results showed that the HIE/n sample exhibited a lower TPC (37.4 ± 0.8 mg/g of dry extract) compared with the HIE/m sample (69.3 ± 1.1 mg/g of dry extract). This nearly twofold difference supports that seasonal variation is a critical factor influencing TPC in *H. incurva* , with the spring period appearing more favorable for phenolic biosynthesis. Nevertheless, contrasting patterns have been reported in the literature. Some studies have observed relatively stable polyphenol levels throughout the year (Ismail et al. 2023; Maribu et al. 2025), whereas Sanz et al. (2023) reported seasonal fluctuations with peak TPC levels occurring between February and May, and again between October and November. In contrast, Kamal et al. (2023) documented the highest TPC levels during the summer period across all examined seaweed species. Collectively, these findings suggest that the seasonal modulation of phenolic content is species‐ and environment‐dependent.

#### 3.4.2 Bromophenols

Bromophenols (BPs) are common marine secondary metabolites, structurally characterized by the one or more benzene rings substituted with bromine and hydroxyl groups (Barciela et al. 2024). The first marine bromophenols (BPs) were isolated from red algae (Katsui et al. 1967). Red algae are generally recognized as particularly rich sources of BPs (Barciela et al. 2024). Bromophenols are considered complex marine phenolics and biosynthesized via substrate bromination reactions involving hydrogen peroxide and bromide ions by vanadium‐dependent bromo‐ or iodoperoxidases (Liu et al. 2011; Barciela et al. 2024). These enzymes exhibit notable stability under stressful environmental conditions, allowing their activity to persist even after the death and partial decomposition of the seaweed host (Mandrekar et al. 2019). Bromophenols isolated from red algae have demonstrated a broad spectrum of biological activities (Mandrekar et al. 2019; Dong et al. 2020; Nagahawatta et al. 2024; Barciela et al. 2024). Nevertheless, despite their biological potential, relatively few studies have focused on the isolation and detailed characterization of these compounds, a limitation that has been largely attributed to their low natural abundance in seaweeds (Lomartire et al. 2021).

The discovery of the bromophenols compounds in *H. incurva* dates back to 1956, when Augier and Mastagli isolated the first sulfated bromophenol from this species and characterized it as C_6_ (SO_3_K)_2_ Br (OH)_2_ COOH (Augier and Mastagli 1956). This compound was later reported to exhibit antioxidant and antimicrobial activities (Liu et al. 2011), as well as an enzymatic inhibitory effect, particularly against human carbonic anhydrases (Dong et al. 2020). Subsequently, Chantraine et al. (1973) identified two additional bromophenols from *H. incurva* : 3,5‐dibromo‐4‐hydroxyphenylacetic acid and 3,5‐dibromo‐4‐hydroxyphenylpyruvic acid. Although limited biological data are available for these compounds, 3,5‐dibromo‐4‐hydroxyphenylacetic acid has demonstrated strong DPPH radical scavenging activity, suggesting antioxidant potential (Liu et al. 2022). Afterward, in 1974, Glombitza et al. reported the presence of 3‐brom‐4,5‐dihydroxybenzyl alcohol and 3‐brom‐4‐hydroxybenzyl alcohol (Glombitza et al. 1974). 3‐brom‐4,5‐dihydroxybenzyl alcohol was also isolated from the tropical green alga *Avrainvillea nigricans* and exhibited significant anticancer activity in tissue culture (Colon et al. 1987). This compound showed cytotoxicity toward KB cells with an IC_50_ value of 8.9 μg/mL (47 μM), exceeding the activity of avrainvilleol (IC_50_ of 10–100 μg/mL) and 5‐Hydroxyisoavrainvilleol, which was inactive. In the same study, it demonstrated broad‐spectrum antimicrobial effects against *Bacillus subtilis, Staphylococcus aureus, Pseudomonas aeruginosa, Escherichia coli, Serratia marcescens*, and *Candida albicans*. This compound was also the most effective and showed the broadest spectrum of activity. Additionally, 3‐brom‐4,5‐dihydroxybenzyl alcohol isolated from the red alga *Polysiphonia morrowii* showed hypoglycemic potential through α‐glucosidase inhibition (IC_50_ of 100 μM; *K*_*i*_ value of 81 μM) and inhibited rat intestinal sucrase and maltase with IC_50_ values of 3.6 and 4.8 mM, respectively (Kurihara et al. 1999). In their study, structure‐activity relationship analyses revealed that methylation of phenolic hydroxyl groups markedly reduced hypoglycemic activity, whereas increased bromination enhanced both enzymes' inhibitory effects. Similarly, for anticancer activity, higher degrees of bromination have been reported to strengthen biological efficacy (Liu et al. 2011). Further bromophenols were identified from *H. incurva* in subsequent studies, including 2,6‐dibromo‐3,5‐dihydroxyphenylacetic acid from acidified ethanolic extracts (De Nanteuil and Mastagli 1981) and, more recently, 3‐bromo‐4,5‐dihydroxyphenylethanol sulfate (Álvarez‐Gómez et al. 2019). The latter compound has also been isolated together with other phenylethanol sulfate bromophenols from the red alga *Rhodomela confervoide* and demonstrated anticancer activity against five human cancer cell lines including colon cancer (HCT‐8), ovarian cancer (A2780), stomach cancer (BGC‐823), hepatoma (Bel7402), and lung adenocarcinoma (A549) (Ma et al. 2006). Based on these results, in contrast to bromination, the presence of a sulfate group does not appear to significantly enhance biological activity.

To the best of our knowledge, detailed biological evaluations of the bromophenols identified in *H. incurva* have not been yet reported. However, the structural similarities between these compounds and other bromophenols known to exhibit diverse biological properties (Liu et al. 2011; Dong et al. 2020) underscore the need to elucidate their specific bioactivities and underlying mechanisms of action. Moreover, comprehensive investigation into their pharmacological profiles, toxicity, and safety are required. In addition, more extensive chemical characterization is necessary to fully resolve the bromophenolic profile of this species.

#### 3.4.3 Isoflavones

Isoflavones are a class of polyphenolic compounds belong to the flavonoid family and are characterized by a diphenylpropane structure (Jung et al. 2020). Structurally, they share a common 3‐phenylchromen‐4‐one backbone, which is frequently modified through O‐substitution, glycosylation, and/or prenylation (Esch and Lehmann 2021). Isoflavones are widely known as phytoestrogens and have attracted significant interest due to their reported health promoting properties (Yari 2022). A study conducted by Klejdus et al. (2010) investigated the presence of isoflavones in various seaweeds including *H. incurva*. To date, this study appears to be the first and only report identifying isoflavones in *H. incurva* . Their results revealed the presence of eight isoflavones in *H. incurva* , with ononin begin the most abundant (50.80 ng/g), following by sissotrin (36.75 ng/g), biochanin A (27.68 ng/g), formononetin (24.76 ng/g), genistein (20.90 ng/g), genistin (18.70 ng/g), daidzein (14.19 ng/g), and daidzin (7.19 ng/g).

The authors did not discuss the differences among species or the distribution of these compounds within individual species. Indeed, *H. incurva* typically inhabits exposed coastal and subtidal rocky habitats, where it is frequently subjected to multiple environmental stressors, including strong hydrodynamic forces, high light and UV radiation, fluctuations in temperature and salinity, and periodic nutrient limitation. Such conditions are known to induce physiological stress in macroalgae, often triggering the activation of secondary metabolic pathways. Consequently, the accumulation of isoflavones in *H. incurva* may represent an adaptive response to these stressful environments, contributing to cellular protection and stress tolerance. The observed isoflavone distribution in *H. incurva* , with ononin and sissotrin predominating, likely reflects species‐specific metabolic preferences favoring methylated and glycosylated isoflavones. These compounds represent stable storage forms produced through active O‐methylation and glycosylation, a process that enhances chemical stability and facilitates sequestration under fluctuating marine environmental conditions. Such metabolic features are consistent with stress adaptation strategies in macroalgae; however, targeted biosynthetic studies are required to confirm these hypotheses.

It is worth mentioning that, in their study *H. incurva* was richer in isoflavones than several other evaluated species ( *Hypnea spinella* , *Sargassum vulgare* , *Undaria pinnatifida, Porphyra* sp., *Spongiochloris spongiosa*, *Scenedesmus*, and *Nostoc 1*). Indeed, ononin (Formononetin 7‐O‐glucoside), the predominant isoflavone detected exhibits a broad range of biological activities, as comprehensively reviewed by Bhuia et al. (2023). The chemical structures of the eight identified isoflavones in *H. incurva* are presented in Figure 3.

#### 3.4.4 Mycosporine‐Like Amino Acids

Mycosporine‐like amino acids (MAAs) are low molecular weight secondary metabolites (< 400 to < 500 Da) (Parailloux et al. 2020; Vega et al. 2023), that function primarily as natural ultraviolet absorbing compounds (Sun et al. 2020). To date, MAAs have been identified in more than 570 species of marine macroalgae, predominantly within the Rhodophyta (Sun et al. 2020), and with over 30 structurally distinct of MAAs reported (Vega et al. 2023). Structurally, MAAs are characterized by cyclohexenone or cyclohexenimine core conjugated to an amino acid or amino alcohol, with structural diversity arising from substituent groups (Chrapusta et al. 2017). These chromophoric systems confer strong UV absorption, with maxima typically ranging from 310 to 362 nm depending on the specific MAA structure (Punchakara et al. 2023). Beyond photoprotection, MAAs have been associated with several biological activities (Punchakara et al. 2023). The MAA profile of *H. incurva* has been investigated in a limited number of studies. Alvarez‐Gómez et al. (2016) quantified total MAAs and six individual MAA compounds (palythinol, palythine, asterina‐330, shinorine, porphyra‐334, and mycosporine‐serinol) in four extracts of *H. incurva* prepared with solvents of different polarity (H_2_O, 20% MeOH, 50% EtOH, and 100% EtOH). Total MAA contents were (0.2 ± 0.1, 0.1 ± 0.0, 0.1 ± 0.0, and 0.04 ± 0.0 mg g^−1^ DW, respectively), indicating a clear influence of solvent polarity on extraction efficiency. Among the identified MAAs, palythinol was the predominant compound across most extracts. The relative proportion of palythinol in the four extracts was 50.3% ± 6.1%, 38.1% ± 19.6%, 64.4% ± 8.4%, and 58.7% ± 2.1%, respectively. Palythine accounted for 10.8% ± 9.0%, 21.6% ± 10.3%, 18.0% ± 8.1%, and 9.0% ± 2%, respectively, while shinorine represented 16.4% ± 8.8%, 28.2% ± 12%, 17.6% ± 6.5%, and 32.4% ± 4.2%, respectively. Porphyra‐334 was detected only in the H_2_O extract (22.5% ± 16.9%). In contrast, asterina‐330 and mycosporine‐serinol were not detected in any of the examined extracts. The authors did not discuss the influence of solvent polarity on MAA composition nor the predominance of palythinol in *H. incurva* . The observed dominance of palythinol and palythine over other MAAs may therefore indicate species specific regulation of MAA biosynthetic pathways, favoring compounds that provide broad photoprotection with lower metabolic cost. Palythinol and palythine are biosynthetically related MAAs that likely originate from the same pathway, differing by late‐stage enzymatic modifications. Conversely, the absence or low abundance of other MAAs (e.g., asterina‐330 or mycosporine‐serinol) suggests that MAA composition in *H. incurva* is tightly regulated and may vary with environmental conditions such as light regime, nutrient availability, and seasonality. Nevertheless, targeted biosynthetic and ecological studies are required to confirm these hypotheses. Supporting the biological relevance palythinol, along with shinorine, palythine, asterina‐330, and porphyra‐334 isolated from the red alga *Palmaria palmata* (dulse) reported to inhibit murine skin melanoma (cell line B16‐F1) in a dose‐dependent manner the proliferation (Yuan et al. 2009).

In a subsequent study, Álvarez‐Gómez et al. (2019) demonstrated MAA accumulation in *H. incurva* is strongly influenced by radiation conditions, inducing marked temporal changes in relative abundance of individual MAAs, with some compounds progressively replaced by others. Samples were cultured for 14 days under PAR (400–700 nm) or PAB (280–700 nm) in nutrient enriched seawater. The total MAA content increased significantly during the first four days, with a stronger response observed under PAB treatment, followed by a marked decline over time. The initial MAA profile in *H. incurva* included asterina‐330, palythine, and palythinol, with asterina‐330 accounting for approximately 90% of total MAAs. However, by day 14 shinorine became the dominant in both treatments, while palythinol was no longer detected. Asterina‐330 and palythinol levels increased under PAB conditions compared to PAR, whereas shinorine and porphyra‐334 were reduced under PAB exposure. This differential response likely reflects functional specialization among MAAs, whereby PAB exposure promotes accumulation of more photostable and efficient UV screening compounds such as asterina‐330 and palythinol, while reducing levels of MAAs with lower stability or precursor roles, such as shinorine and porphyra‐334. Notably, radiation treatments enhanced total MAA accumulation, with total MAAs exceeding 0.50 ± 0.00 mg g^−1^ DW after 4 days under PAB treatment, compared with the earlier study (Alvarez‐Gómez et al. 2016). The presence of asterina‐330 in this study which was reported as the predominant and its absence in an earlier investigation (Alvarez‐Gómez et al. 2016) may reflect the highly inducible and environmentally responsive nature of this MAA. This difference cannot be explained by solvent polarity, as identical extracts were used in both studies, nor by geographical origin, since samples were collected from the same site. Asterina‐330 is known to accumulate under elevated UV exposure and may represent a transient stress‐response metabolite. Seasonal variation, differences in radiation regime, and rapid metabolic turnover could therefore account for its detection under controlled PAB conditions and its absence in field collected samples. As neither study reported sampling season, definitive conclusions cannot be drawn.

In another investigation, Parailloux et al. (2020) identified several MAAs in *H. incurva*, including palythine, mycosporine‐glycine, palythene/Usujirene, asterina‐330, palythinol, shinorine, and porphyra‐334, as well as an unknown MAA with molecular formula C_14_H_22_O_8_N_2_ (Parailloux et al. 2020). However, this study did not include quantitative analysis, limiting the comprehensive assessment of MAA abundance in *H. incurva* . Consequently, further research is required to quantify the relative levels of individual MAAs and to better characterize their distribution. Overall, studies on MAAs in *H. incurva* remain scarce, underscoring the need for additional investigations to elucidate their chemical diversity and explore their potential biological and therapeutic relevance. The chemical structures of the MAAs identified in *H. incurva* are presented in Figure 3.

#### 3.4.5 Carbohydrate Profiling

Red seaweeds are recognized by a high carbohydrates content (Tavares et al. 2023), associated with specific chemical structures that differ from those of terrestrial plants and other algal groups (Rupérez 2002). In particular, they are rich in the sulfated galactans, including carrageenan, agarose, and agar, with a wide range of biological properties (Cian et al. 2015; Raúl et al. 2015; Tavares et al. 2023; Udayakumar et al. 2024). Accordingly, comparative studies have shown that extracts prepared from *H. incurva* are particularly rich in carbohydrates. Alvarez‐Gómez et al. (2016) quantified the total carbohydrate percentage from four extracts prepared using solvents of different polarity (H_2_O, 20% MeOH, 50% EtOH, and 100% EtOH). The total carbohydrate contents were 7.1% ± 3.7%, 16.1% ± 3.3%, 6.6% ± 3.3%, and 8.5% ± 0.9%, respectively, clearly demonstrating the influence of solvent polarity on carbohydrate extraction efficiency. Notably, the methanolic extract exhibited higher carbohydrate levels compared to the several studied seaweeds ( *Gelidium pusillum* , *Gelidium corneum* , *Porphyra umbilicali*, *Gracilariopsis longissim*, *Hydropuntia cornea* , *Ulva rotundata* , and *Ulva rotundata* ).

The impact of geographical origin on carbohydrate content was assessed by Nunes et al. (2020), who analyzed *H. incurva* samples collected from two stations (Abas do Rio and Calhau da Serra de For). Total carbohydrate contents were 34.70 ± 4.62 and 33.35 ± 4.92 g.100 g^−1^ dw, respectively. As also observed for total phenolic content (TPC), the sample collected from the AR station exhibited slightly higher levels.

In another survey, temperature was also shown to affect carbohydrate levels. Plaza et al. (2010) evaluated sugar content following subcritical water extraction at 100°C and 200°C and reported glucose concentrations of 6.71 ± 0.28 and 11.93 ± 0.76 g.100 g^−1^, respectively, indicating a nearly twofold increase with higher extraction temperature. This trend contrasts with other algae (e.g., *Porphyra* spp.) and terrestrial plants (*Rosemary*, *Thyme*, and *Verbena*), in which glucose levels decreased with increasing temperature. Notably, a comparable temperature‐dependent trend was also reported for total phenolic content (TPC) in the same study.

In addition to the parameters discussed above, seasonal variation may also influence total carbohydrate, although further investigations are required to clarify this effect. Several studies have reported that carbohydrate synthesis in macroalgae tends to increase during colder periods, from October to March/April, and to decrease during warmer months between May and July (Sanz et al. 2023). Consistently, Ismail et al. (2023) reported the highest carbohydrate levels during winter and autumn. Seasonality has also been shown to affect monosaccharide composition, with xylose levels peaking in February and galactose and glucose reaching maximum levels in August (Maribu et al. 2025). In addition, further chemical characterization studies are needed to elucidate the carbohydrate composition of *H. incurva* .

#### 3.4.6 Protein Profiling

Algae are regarded as valuable protein sources, occurring various benefits due to their nutritional value with exceptional amino acid profiles compared to animal and land plant sources (Camila et al. 2023; Thiviya et al. 2022). Red algae are characterized by higher protein contents than brown and green algae (Gamero‐Vega et al. 2020), and are increasingly recognized as functional food ingredients. Phycobiliproteins represent their major protein fraction, accounting for up to 50% of the total protein content (Cian et al. 2015). *H. incurva* has been identified as a prominent source of protein, with reported protein contents of 9.41 ± 0.19 and 7.69 ± 0.04 g 100 g^−1^ DW in samples collected from Abas do Rio (AR) and Calhau da Serra de For (CSF), respectively. As also observed for total phenolic content (TPC) and carbohydrate, the sample collected from the AR station contained higher levels. Indeed, both sites are located on Porto Santo Island (Madeira Archipelago); however, AR is situated on the eastern coast, whereas CSF lies on the southern coast, suggesting that local environmental conditions may contribute to the observed differences. Consequently, further investigation of the environmental characteristics of both stations would be valuable to better understand their influence on protein accumulation. In that study, *H. incurva* contains approximately three to fourfold higher protein than the red alga *Corallina officinalis* and several brown algae ( *Cystoseira compressa* , *Cystoseira humilis*, *Cystoseira usneoides*, *Dictyota dichotoma* , *Halopteris filicina* , *Halopteris scoparia* , *Lobophora variegata* , *Padina pavonica*, *Sargassum vulgare* ), as well as the green alga *Ulva* sp.

The influence of temperature on protein and amino acid content was examined by Plaza et al. (2010), using subcritical water extraction. In *H. incurva* amino acid content decreased significantly with increasing temperature from 29.37 ± 1.12 μmol/g d.m at 100°C to 14.97 ± 0.66 μmol/g d.m at 200°C, respectively. This behavior contrasts with the temperature‐dependent increase observed for carbohydrates and total phenolic content, suggesting thermal degradation or reduced stability of amino acids at elevated temperatures. Additionally, *H. incurva* exhibited higher amino acid contents at both temperatures compared with all evaluated samples, including the microalga *Chlorella vulgaris* , macroalgae *Cystoseira abies‐marina*, *Sargassum muticum* , *Porphyra* spp., *Sargassum vulgare* , and *Undaria pinnatifida*, and terrestrial plants *Verbena, Thyme*, and *Rosemary*, which exhibited the lowest contents. This pronounced amino acid richness highlights the need for detailed qualitative and quantitative analyses to fully characterize the amino acid profile of *H. incurva*. In addition, *H. incurva* showed a high protein percentage (24.3% ± 0.3%), exceeding those of *Cystoseira abies‐marina*, *Sargassum vulgare* , *Undaria pinnatifida*, *Sargassum muticum* , *Rosemary*, *Thyme*, and *Verbena*. However, *Chlorella vulgaris* and *Porphyra* spp. exhibited a higher protein percentage than *H. incurva*, despite having lower amino acid contents, indicating qualitative differences in protein composition among species.

Further evidence of the protein profile of *H. incurva* was provided by Terriente Palacios et al. (2022), who quantified protein content and protein isolates in enzymatic protein hydrolysates (EPH). Protein contents of 13.5 ± 1.87 g. 100 g^−1^ DW in raw material and 7.41 ± 1.74 g. 100 g^−1^ DW in protein isolates were reported, with a protein recovery of 54.9% ± 2.39%. In that study, *H. incurva* contained higher protein contents than other studied red algae (*Centroceras clavulatum, Plocamium cartilagineum, Gelidium corneum, Mastocarpus stellatus*, and *Chondrus crispus* ). In the same study, *H. incurva* were characterized by elevated levels of peptides and bioactive amino acid derivatives. Peptide and sum of hydrophobic amino acid (SHA) contents reached 2.81 ± 0.04 and 47.0 ± 2.65 mg Glu eq/mL, respectively, while, taurine, homotaurine, and γ‐aminobutyric acid (GABA) were 0.04 ± 0.01, 0.02 ± 0.01, and 0.37 ± 0.03 g/100 g d.w, respectively. Among all the red seaweeds examined in that study, *H. incurva* exhibited highest peptide and SHA contents, supporting its potential as a promising source of neuroprotective and functional protein‐derived compounds.

#### 3.4.7 Lipid Profiling

Although red algae typically contain low total lipid levels, they are recognized for the high quality of their lipid fraction, especially their abundance of polyunsaturated fatty acids (PUFAs) with documented nutritional and bioactive relevance (Gamero‐Vega et al. 2020). Nunes et al. (2020) evaluated lipid content of *H. incurva* samples collected from two sites Abas do Rio (AR) and Calhau da Serra de For (CSF). The total lipid contents ranged from 3.83 ± 0.35 to 5.05 ± 0.86 g 100 g^−1^ dw, respectively. In contrast to the trends observed for total phenolic content (TPC), carbohydrates, and proteins, lipid levels were higher in the CSF samples than in those from AR and may reflect the distinct physiological roles and regulatory mechanisms governing metabolism in macroalgae. Unlike phenolics and proteins, which often accumulate in response to environmental stress as protective or defensive compounds, lipids primarily contribute to membrane structure, fluidity, and energy storage. Consequently, lipid biosynthesis may be favored under more stable conditions, potentially associated with reduced hydrodynamic stress or differences in light and temperature regimes at the CSF site. In contrast, the AR site may promote the accumulation of phenolics and proteins as part of stress‐related adaptive responses. However, detailed environmental characterization and lipid class‐specific analyses are required to confirm these hypotheses.

In contrast, Alvarez‐Gómez et al. (2016) reported no total lipid content in *H. incurva* extracts. However, in a subsequent study examining the influence of ultraviolet radiation (UVR), the same authors reported an initial lipid content of approximately 4% DW prior to UV exposure (Álvarez‐Gómez et al. 2019). Lipid levels increased significantly after 4 days under both PAR and PAB treatments, with nearly a twofold increase under PAB. Although lipid content decreased under PAB on day 8, a marked increase was observed by day 14, reaching approximately 9% DW, particularly under PAB conditions.

In a broader lipidomic survey conducted by Galindo et al. (2021) on14 macroalgal species, including *H. incurva*, a total lipid content of 1.2% ± 0.1% DW was reported. Detailed lipid class analysis revealed that neutral lipids were the predominant fraction in *H. incurva* (41.1% ± 5.4%), followed by total polar lipids (29.3% ± 4.6%), free fatty acids (11.1% ± 1.1%), and phytosterols (14.6% ± 1.1%). The major polar lipid classes comprised monogalactosyldiacylglycerols (5.6% ± 0.4%), digalactosyldiacylglycerols (6.7% ± 0.3%), phosphatidylcholine (6.2% ± 1.3%), phosphatidylserine + phosphatidylinositol (1.3% ± 0.6%), and sulfoquinovosyldiacylglycerol + phosphatidylethanolamine (7.7% ± 1.8%). Compared with other red algae screened in the study ( *Corallina officinalis* and *Asparagopsis taxiformis* ), *H. incurva* exhibited higher proportions of galactolipids, phosphatidylcholine, and total polar lipids.

**FIGURE 2:**
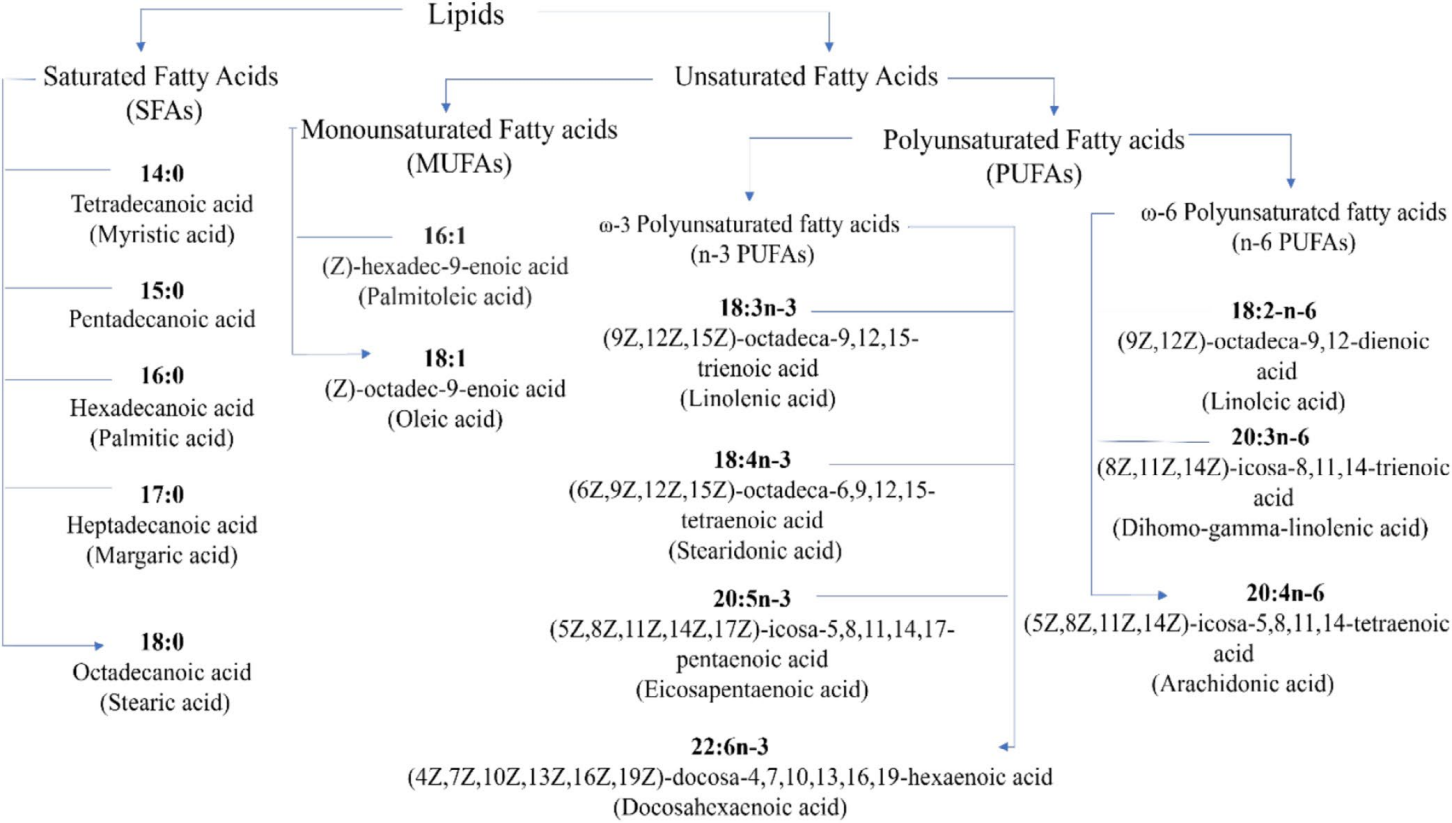
Graphical chart of the fatty acids identified in *H. incurva* .

**FIGURE 3:**
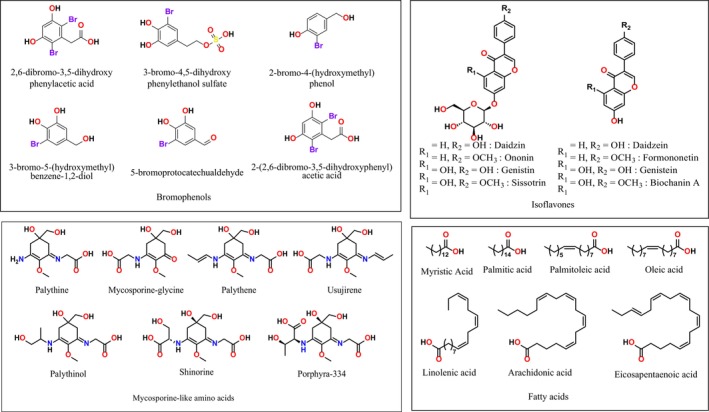
Chemical structures of bromophenols, mycosporine‐like amino acids, isoflavones, and the major fatty acids identified in *H. incurva*.

In the same study the fatty acid profiling showed that saturated fatty acids (SFAs) accounted for 42.3% ± 1.0% of total fatty acids, dominated by palmitic acid (16:0; 32.0% ± 0.6%). Monounsaturated fatty acids (MUFAs) represented 20.0% ± 0.3%, mainly oleic acid (18:1; 12.9% ± 0.3%). Total n‐6 PUFAs accounted for 14.2% ± 0.8%, largely composed of arachidonic acid (20:4n‐6; 11.7% ± 0.8%), while total n‐3 PUFAs reached 21.6%, dominated by α‐linolenic acid (18:3n‐3; 10.9% ± 0.6%) and eicosapentaenoic acid (EPA, 20:5n‐3; 9.3% ± 0.6%). Notably, *H. incurva* exhibited higher EPA content (9.3%) than other red algae such as *Corallina officinalis* (2.2% ± 0.4%) and *Asparagopsis taxiformis* (3.4% ± 0.6%). The calculated nutritional indices further supported its health relevance, with favorable n‐6/n‐3 (1.1 ± 0.1), DHA/EPA (0.1 ± 0.0), and ARA/EPA (1.3 ± 0.1) ratio. Additionally, assessment of lipid nutritional quality using atherogenicity (AI) and thrombogenicity (TI) indices revealed values of 1.1 ± 0.1 and 0.5 ± 0.0, respectively (Galindo et al. 2021), indicating a low risk for cardiovascular disorders. All screened seaweeds exhibited n‐6/n‐3 ratios below the threshold recommended by the World Health Organization (< 10), with *H. incurva* displaying one of the most favorable profiles. Overall, the lipid composition of *H. incurva* , characterized by high PUFA content, elevated EPA levels, favorable nutritional indices, and abundant bioactive lipid classes, highlights its potential as a valuable nutraceutical resource for cardiovascular health. However, to date, comprehensive fatty acid characterization has been limited to a single study (Galindo et al. 2021) underscoring the need for further investigations to fully elucidate the lipidomic profile of this species. All identified fatty acids are presented as a graphical chart in Figure 2, while the chemical structures of the major fatty acids identified in *H. incurva* are shown in Figure 3.

#### 3.4.8 Pigments

Rhodophyceae are characterized by the presence of chlorophyll a and d together with accessory pigments such as phycobiliproteins and carotenoids. These pigments confer the characteristic reddish coloration of red algae and enable efficient light harvesting in blue‐green wavelengths, allowing growth at depths of up to 200 m (Cian et al. 2015), as well as supporting their adaptation to fluctuating and often stressful marine environments. Red seaweeds are particularly rich in pigments, with phycobiliproteins representing the only water‐soluble pigments in this group and accounting for up to 50% of total protein content (Cian et al. 2015; Thiviya et al. 2022). Phycobiliproteins are classified into allophycocyanin (APC), phycocyanin (PC), phycoerythrocyanins, and phycoerythrin (PE), the latter being the dominant pigment in most Rhodophyta (Thiviya et al. 2022).

A geographical comparison conducted by Nunes et al. (2020) assessed pigment contents *in H. incurva* collected from two sites Abas do Rio (AR) and Calhau da Serra de For (CSF). Chlorophyll a and total carotenoids were significantly higher in AR samples (11.38 ± 0.43 and 6.59 ± 0.42 mg g^−1^ DW, respectively) than in CSF samples (7.07 ± 0.43 and 4.58 ± 0.25 mg g^−1^ DW). In contrast, phycobiliproteins were more abundant in CSF samples, with APC and PC reaching 0.33 ± 0.03 and 0.14 mg g^−1^ DW, respectively, compared with 0.12 ± 0.06 and 0.010 ± 0.01 mg g^−1^ DW in AR, respectively. Phycoerythrin contents were (0.82 ± 0.05 mg g^−1^ DW in AR and 0.85 ± 0.07 mg g^−1^ DW in CSF samples). These results suggest that AR conditions favor chlorophyll a and carotenoid biosynthesis, whereas CSF conditions promote phycobiliprotein accumulation, likely reflecting site‐specific light regimes and environmental constraints. These site dependent pigment patterns are consistent with the biochemical trends observed for lipids and proteins. The higher chlorophyll a and carotenoid contents in AR samples parallel the increased lipid levels, reflecting the close association of these pigments with photosynthetic membranes and galactolipids. In contrast, the higher phycobiliprotein contents in CSF samples align with greater protein allocation, suggesting site‐specific metabolic adjustment driven by local light conditions and environmental constraints. *H. incurva* collected from CSF site exhibited the highest contents of all three phycobiliprotein classes among the red algae analyzed in this study. This finding underscores the strong nutritional and biological potential of *H. incurva* , given that phycobiliproteins are well known for their broad spectrum of biological activities (García‐Gómez et al. 2025).

Cultivation effects were evaluated by Vega et al. (2020), who compared wild‐collected and IMTA‐cultivated *H. incurva* . Chlorophyll a and carotenoid contents showed minor differences between conditions (3.8 and 4 mg Chl *a* g^−1^ DW, respectively). Whereas phycoerythrin increased markedly after cultivation, reaching 24.0 mg g^−1^ DW compared to 3.8 mg g^−1^ DW in wild samples. These values were higher than those reported for other studied red algae supporting *H. incurva* as an exceptional source of phycobiliproteins.

The influence of UV radiation was investigated by Álvarez‐Gómez et al. (2019), who reported that *H. incurva* displayed the highest proportion of phycobiliproteins (up to 57%) among tested species. Chlorophyll a and phycocyanin (PC) contents increased progressively over time under both PAR and PAB conditions, whereas carotenoids were the only pigments significantly influenced by both radiation regime and exposure time; phycobiliproteins were primarily time‐dependent. Under PAR conditions, carotenoid content increased steadily from 0.19 ± 0.01 mg g^−1^ DW (day 4) to 0.28 ± 0.01 mg g^−1^ DW (day 8), reaching 0.30 ± 0.03 mg g^−1^ DW by day 14. Under PAB conditions, carotenoids showed a slight decrease from 0.26 ± 0.01 to 0.24 ± 0.01 mg g^−1^ DW between day 4 and day 8, followed by an increase to 0.30 ± 0.02 mg g^−1^ DW at day 14. Phycoerythrin (PE) content decreased initially under both radiation regimes, followed by a marked increase by day 14, reaching 2.41 ± 0.30 mg g^−1^ DW under PAR and 2.37 ± 0.47 mg g^−1^ DW under PAB. Phycocyanin content increased continuously under both treatments, from 0.25 ± 0.03 to 0.44 ± 0.04 mg g^−1^ DW under PAR and from 0.26 ± 0.06 to 0.44 ± 0.08 mg g^−1^ DW under PAB between day 4 and day 14. Notably, whereas chlorophyll variations in other red seaweeds were influenced by both light and time, chlorophyll a change in *H. incurva* were solely time‐dependent. Moreover, *H. incurva* was the only species to exhibit a significant combined effect of light regime and time on carotenoid content.

Similarly Güenaga Unzetabarrenechea (2011) reported detectable pigments in *H. incurva* , with phycoerythrin and chlorophyll *a* levels of 0.03 ± 0.00 and total carotenoids of 0.08 ± 0.00 mg g PS^−1^. No chlorophyll b was detected, confirming the typical Rhodophycean pigment profile of this species. The authors further reported that *H. incurva* exhibited higher chlorophyll and carotenoid contents than other screened red algae (*Dermocorynus dichotomus*, *Hypnea spinella* , *Laurencia majuscula*, and *Pterocladiella capillacea*). Collectively, these findings demonstrate that *H. incurva* is particularly rich in photosynthetic and accessory pigments, underscoring its strong ecological adaptability and highlighting its potential as a valuable natural source of pigments for nutraceutical and biotechnological applications.

#### 3.4.9 Phytohormones

Plant growth regulators (PGRs) are also synthesized by seaweeds and in some cases occur at higher levels in algae than in land plants (Shanab and Shalaby 2021). Algal species are known to enhance PGR accumulation in response to abiotic and biotic stressors. For *H. incurva* , only a single study has addressed this sense. Yalçın et al. (2023) quantified several PGRs in seaweeds, including *H. incurva* collected from the southern coast of Turkey (Antalya), using LC–MS/MS analysis. Quantitative analysis revealed concentrations of 11.80 ± 0.55 ng g^−1^ (indole‐3‐acetic acid), 1.93 ± 0.045 ng g^−1^ (6‐benzylaminopurine), 0.77 ± 0.01 ng g^−1^ (kinetin), 0.49 ± 0.02 ng g^−1^ (abscisic acid), and 0.041 ± 0.00 ng g^−1^ (zeatin), while gibberellic acid was not detected. Indole‐3‐acetic acid was the predominant PGR in *H. incurva* and has been reported to hold diverse biological effects (Shaheen et al. 2025). Notably, *H. incurva* exhibited higher levels of 6‐benzylaminopurine, indole‐3‐acetic acid, and abscisic acid compared to other screened red seaweeds, including *Polysiphonia scopulorum* , *Ellisolandia elongata*, and *Gracilaria bursa‐pastoris* . Compared to terrestrial plants, algal hormone research remains in its early stages, underscoring the need for further studies focused on chemical characterization and functional roles of phytohormones in algae. For *H. incurva* , additional investigations considering factors such as seasonality, developmental stage, and geographical distribution are required to fully elucidate its phytohormonal profile. The chemical structures of the phytohormones characterized in *H. incurva* are presented in Figure 3.

#### 3.4.10 Mineral Content

Owing to their high bioadsorptive and bioaccumulative capacities, seaweeds can contain mineral concentrations up to 10–100 times than those of land plants (Circuncisão et al. 2018). They are frequently regarded as “superfoods” and are considered promising candidates for nutraceutical development (Circuncisão et al. 2018). They are rich in magnesium and provide appreciable amounts of essential trace elements, including manganese, zinc, iodine, and copper (Gamero‐Vega et al. 2020). To date, the study conducted by Nunes et al. (2020) appears to be the only investigation reporting the mineral profile of *H. incurva* . Based on samples collected from two coastal stations, Abas do Rio (AR) and Calhau da Serra de Fora (CSF), total mineral contents reported 48.88 ± 1.45 for AR and 47.88 ± 0.67 g 100 g^−1^ DW CSF. Notably, among all screened species, *H. incurva* exhibited a higher mineral content than several brown algae ( *Cystoseira compressa* , *C. humilis* , *C. usneoides*, *Dictyota dichotoma* , and *Sargassum vulgare* ), the red alga *Asparagopsis taxiformis* , and green algae ( *Ulva intestinalis* and *Ulva* sp.). Furthermore, mineral levels varied considerably among species even when collected from the same site, highlighting the importance of species‐specific accumulation capacity. Overall, the substantial mineral content of *H. incurva* underscores its potential as a valuable nutritional resource. However, comprehensive mineral profiling, including trace and potentially toxic elements, as well as investigations into seasonal variability, are required to fully elucidate its nutritional value and health implications, as mineral composition in macroalgae is known to vary with both season and species (Kamal et al. 2023).

## 4 Biological Activities

Beyond its phytochemical profile, *H. incurva* has attracted considerable scientific interest due to its broad range of reported biological activities. This section provides a comprehensive overview of the biological properties attributed to *H. incurva* , as summarized in Table 3.

**TABLE 3:** Summary of the reported biological activities of *H. incurva* .

Strains used		Extracts	Key results	Country	References
Antibacterial activity					
*Staphylococcus aureus*		DCM/MeOH (50:50)	MIC = 10–15 mm	Oualidia, Morocco	(Farid and Etahiri 2009)
*Bacillus cereus*			MIC = 15 mm		
*Escherichia coli* .			Inactive		
*Clostridium sporogenes*		MeOH	MIC < 15 mm	El‐Jadida, Morocco	(Oumaskour et al. 2013)
		DCM/MeOH (50:50)	MIC < 15 mm		
*Staphylococcus aureus*			MIC > 15 mm		
*Bacillus cereus*					
*Staphylococcus aureus ssp aureus*		Acetone			
*Pseudomonas Aeruginosa*		EtOH		Ain Franin, Algeria	(Khelil‐Radji et al. 2017)
		Water	Inactive		
*Staphylococcus aureus*		EtOH	MIC = 18 mm		
		Water	Inactive		
*Bacillus subtilis*		EtOH	MIC = 10 mm		
		Water	Inactive		
*Escherichia coli*		EtOH	MIC = 10 mm		
		Water	Inactive		
*Bacillus subtilis*		MeOH	MIC > 15 mm	Canary Islands, Spain	(González del Val et al. 2001)
*Enterococcus faecium*			Inactive		
*Staphylococcus aureus*					
*Pseudomonas aeruginosa*					
*Serratia marcescens*					
Antimycobacterial activity					
*Mycobacterium tuberculosis*		Combined crude extract (i‐PrOH + CHCl_3_: MeOH)	MIC = 256 μg/mL	Kimmeridge Dorset, UK	(Allmendinger et al. 2010)
		Isoniazid (reference)	MIC = 0.1 μg/mL		
Leishmanicidal activity					
*Leishmania donovani*		Combined crude extract (i‐PrOH + CHCl_3_: MeOH)	IC_50_ = 16.5 μg/mL		
		Miltefosine (reference)	IC_50_ = 0.2 μg/mL		
Trypanocidal activity					
*Trypanosoma brucei rhodesiense*		Combined crude extract (i‐PrOH + CHCl_3_:MeOH)	IC_50_ = 8.1 μg/mL		
		Melarsoprol (reference)	IC_50_ = 0.003 μg/mL		
*Trypanosoma cruzi*		Combined crude extract (i‐PrOH + CHCl_3_: MeOH)	IC_50_ > 90 μg/mL		
		Benznidazole (reference)	IC_50_ = 0.359 μg/mL		
Antifungal activity					
*Saccharomyces cerevisiae*		MeOH	Inactive	Gran, Canary Islands, Spain	(González del Val et al. 2001)
*Candida albicans*					
*Botrytis cinerea*		MeOH	16.8 ± 0.07 mm	Atlantic Ocean, Morocco	(Bahammou et al. 2021)
*Penicillium digitatum*			4.9 ± 0.04 mm		
*Cryptococcus neofomans*		DCM/MeOH (50/50)	< 10 mm	Oualidia, Morocco	(Farid and Etahiri 2009)
*Fusarium oxysporum* f. sp. *Albedinis*		EtOH	48.71%	Ain Franin, Algeria	(Khelil‐Radji et al. 2017)
		Water	61.53%		
*Podosphaera xanthii*		Water	96.8%	Spain	(Roberti et al. 2016)
*Aspergillus fumigatus*		MeOH	Inactive	Canary Islands, Spain	(González del Val et al. 2001).
Molluscicidal activity					
*Biomphalaria glabrata snails*		Water fraction of 500 ppm MeOH extract	100%	Kimmeridge Dorset, UK	(Patel et al. 2008)
		Fraction after PVPP	Inactive		
		LMW			
		HMW			
Antiplasmodial activity					
Stage inhibition assays	*LB*	CHCl_3_: MeOH (3:1 and 1:1)	IC_50_ = 50 μg/mL	Kimmeridge, Dorset, UK	(Spavieri et al. 2013)
		Chloroquine (reference)	IC_50_ = 0.056 μg/mL		
	*LS*	CHCl_3_: MeOH (3:1 and 1:1)	IC_50_ = 28.8 μg/mL		
		Primaquine (Reference)	IC_50_ = 3.4 μg/mL		
Enzyme inhibition assays	PfFabI	CHCl_3_: MeOH (3:1 and 1:1)	IC_50_ = 2.4 μg/mL		
		Primaquine (reference)	IC_50_ = 0.014 μg/mL		
	PfFabZ	CHCl_3_: MeOH (3:1 and 1:1)	IC_50_ = 2.9 μg/mL		
		Triclosan (reference)	IC_50_ = 0.03 μg/mL		
	PfFabG	CHCl_3_: MeOH (3:1 and 1:1)	IC_50_ = 15.9 μg/mL		
		Triclosan (reference)	IC_50_ = 0.30 μg/mL		

Assays		Extract, fraction and control used	Results	Country	References
Antioxidant activity					
DPPH		EtOH/H_2_O (70:30), May	45.6 ± 3.6 mg/g DW	Livorno, Tuscany, Italy	(Vasarri et al. 2020)
		EtOH/H_2_O (70:30), November	82.6 ± 0.5 mg/g DW		
FRAP		EtOH/H_2_O (70:30), May	137.39 ± 14.70 mg/g DW		
		EtOH/H_2_O (70:30), November	24.84 ± 1.82 mg/g/DW		
		MeOH, wild‐collected	24.28 ± 0.03 μg AA mg/DW^−^	Playa de Jimanar, Spain	(Vega et al. 2020)
		MeOH, cultivated	2.30 ± 0.04 μg AA mg/DW^−^		
		EtOH 50%, wild‐collected	53.81 ± 0.22 μg. AA mg/DW^−^		
		EtOH 50%, cultivated	22.10 ± 0.15 μg AA mg/DW		
DPPH		MeOH, wild‐collected	EC_50_ = 2.47 ± 0.01 mg/mL		
		MeOH, cultivated	EC_50_ = 2.54 ± 0.01 mg/mL		
		EtOH 50%, wild‐collected	EC_50_ = 2.62 ± 0.02 mg/mL		
		EtOH 50%, cultivated	EC_50_ = 2.66 ± 0.04 mg/mL		
β‐carotene test		MeOH	EC_50_ = 51.09 ± 0.14 mg/mL	Sidi Bouzid Atlantic coast, Morocco	(Bouhlal et al. 2013)
DPPH			Inactive		
		BHA (reference)	8.38 ± 0.09 μg/mL		
		BHT (reference)	11.43 ± 0.17 μg/mL		
		EPHF	68.5% ± 0.11%	Trasvia, Comillas, Cantabria	(Terriente Palacios et al. 2022)
ABTS			65.7% ± 0.85%		
FRAP		EtOH50%	3.15 μg AA mg^−1^		(Güenaga Unzetabarrenechea 2011)
		MeOH extract	24.28 ± 0.024 μg AA mg^−1^		
			Inhibition % = 93.02%		
			EC_50_ = 2.47 ± 0.01 mg/mL		
		EtOH 50%	EC_50_ = 2.62 ± 0.02 mg/mL		
ABTS		SWE (100°C)	0.355 ± 0.051 mmol TE/g d.m	Las Palmas, Spain	(Plaza et al. 2010)
		SWE (200°C)	1.042 ± 0.017 mmol TE/g d.m		
ORAC_FL_		SWE (100°C)	629.80 ± 68.75 μmoL TE/g		
		SWE (200°C)	1498.75 ± 221.67 μmoL TE/g		
Superoxide radical scavenging capacity		SWE (100°C)	IC_50_ = 7.901 ± 0.16 mg/mL		
		SWE (200°C)	IC_50_ = 5.851 ± 0.07 mg/mL		
DPPH		Crude extract	EC_50_ = 0.154	El Jadida coast, Morocco	(Nmila and Rchid 2018)
		Chloroform extract	EC_50_ = 0.150		
		Isopropanolic extract	EC_50_ = 1.320		
		δ‐Tocopherol (reference)	EC_50_ = 0.260		
ABTS		Acetone/MeOH (1:1, 2 L)	≈55%	Ksar‐sghir, Morocco	(Zbakh et al. 2014)
		Trolox (reference)	≈98%		
β‐carotene test		Water	≈8 μmoL TE/g DW	Tarifa, Cádiz, Spain	(Alvarez‐Gómez et al. 2016)
		20% MeOH			
		50% EtOH			
		100% EtOH	≈7.5 μmoL TE/g DW		
DPPH		Water	≈8.5 μmoL TE/g DW		
		20% MeOH	≈10.5 μmoL TE/g DW		
		50% EtOH	≈10 μmoL TE/g DW		
		100% EtOH			
ABTS		Water	≈7 μmoL TE/g DW		
		20% MeOH			
		50% EtOH			
		100% EtOH	≈6 μmoL TE/g DW		
Anti‐inflammatory and immunomodulatory activities					
In vitro enzyme inhibition	Elastase	DCM/MeOH (50:50)	Inactive	Sidi Bouzid, El Jadida, Morocco	(Oumaskour et al. 2013)
	Phospholipase 2		≈20%		
In vitro RAW 246.7 Macrophage	Proliferation	Polysaccharide	No effect	Canary Islands, Spain	(Abdala Díaz et al. 2011)
	TNF‐α production		No TNF‐α induction		
	IL‐6 production		IL‐6 ≈4 ng/mL at 50 μg/mL		
	NO production		NO ≈16 nM at 50 μg/mL		
Zebrafish (*Danio rerio*, in vivo)		Dietary *H. incurva* (0.25%–1%)	↑ lysozyme activity, ↑ complement activity, ↑ total immunoglobulins (IgM), ↑ antioxidant enzymes, immune‐gene upregulation	Iskenderun Bay coast, Hatay, Turkey	(Hoseinifar et al. 2022)
Antitumoral activity					
In vitro model used	HT29 cell line	Acetone/MeOH (13 μg/mL, 8 h)	IC_50_ = 88.26 μg/mL	Ksar‐sghir, Morocco	(Zbakh et al. 2014)
		Acetone/MeOH (13 μg/mL, 72 h)	IC_50_ = 77.62 μg/mL		
		Acetone/MeOH (200 μg/mL, 48 h)	IC_50_ = 88.26 μg/mL		
		Acetone/MeOH (200 μg/mL, 72 h)	IC_50_ = 77.62 μg/mL		
	L‐6 cell line	Combined crude extract (i‐PrOH + CHCl_3_: MeOH)	IC_50_ = 90 μg/mL	Kimmeridge, Dorset, UK	(Allmendinger et al. 2010)
		EGCG (Reference)	0.004 μg/mL		
		Podophyllotoxin (Reference)			
	Canine C2 cell line	DCM: MeOH	484.8 μg/mL	Morocco coast	(Amminikutty 2021)
Neuroprotective activity					
In vitro enzyme inhibition	AChE	EtOH	12.01% ± 1.51%	Antalya, Turkey	(Kilic et al. 2021)
		Galanthamine	96.02% ± 1.98%		
	BChE	EtOH	9.41% ± 1.82%		
		Galanthamine	82.35% ± 5.36%		

Abbreviations: ABTS, 2,2′‐azino‐bis(3‐ethylbenzothiazoline‐6‐sulfonic acid); AChE, acetylcholinesterase; BChE, butyrylcholinesterase; BHA, butylated hydroxyanisole; BHT, butylated hydroxytoluene; CHCl_3_, chloroform; DCM, dichloromethane; DPPH, 2,2‐diphenyl‐1‐picrylhydrazyl; DW, dry weight; EC_50_, half‐maximal effective concentration; EGCG, (−)‐epigallocatechin gallate; EtOH, ethanol; FRAP, ferric reducing antioxidant power; HMW, high‐molecular‐weight fraction; i‐PrOH, isopropanol; IC_50_, half‐maximal inhibitory concentration; IgM, immunoglobulin M; IL‐6, interleukin‐6; LB, liver stage; LMW, low‐molecular‐weight fraction; MAAs, mycosporine‐like amino acids; MeOH, methanol; MIC, minimum inhibitory concentration; NO, nitric oxide; ORAC‐FL, oxygen radical absorbance capacity–fluorescein; PfFabG, *Plasmodium falciparum* enoyl‐ACP reductase (FabG); PfFabI, *P. falciparum* enoyl‐ACP reductase (FabI); PfFabZ, *P. falciparum* β‐hydroxyacyl‐ACP dehydratase (FabZ); PLA_2_, phospholipase A_2_; PVPP, polyvinylpolypyrrolidone; RAW 264.7, murine macrophage cell line; SWE, subcritical water extract; TE, Trolox equivalents; TNF‐α, tumor necrosis factor‐α.

### 4.1 Antimicrobial Activities

Antibiotic resistance has emerged as a rapidly escalating global public health challenge, severely compromising the effectiveness of conventional antimicrobial therapies. According to the World Health Organization's annual pipeline report, the development of new antimicrobial agents remains insufficient to counterbalance the growing threat posed by resistant pathogens (OMS 2022). Consequently, there is an urgent need to identify new antimicrobial compounds and explore alternative therapeutic strategies. In this context, several studies have demonstrated the notable antimicrobial potential of *H. incurva* against a wide range of microbial strains.

#### 4.1.1 Antibacterial Activity

Numerous researchers have investigated the antibacterial activity of *H. incurva* against both Gram‐negative and Gram‐positive pathogenic strains. Farid and Etahiri (2009) evaluated the potential of 19 seaweeds belonging from different groups, including *H. incurva* . According to their results, dichloromethane‐methanol extract of *H. incurva* exhibited inhibition diameters greater than 15 mm against *Bacillus cereus* , moderate activity (10 and 15 mm) against *Staphylococcus aureus* , and no detectable activity against *Escherichia coli* . Seasonality impact was also investigated, revealing that maximal antibacterial activity across all tested species occurred in spring, which the authors associated with increased salinity. Notably, *H. incurva* showed stronger activity against *B. cereus* than other screened *Asparagopsis armata* , *Corallina officinalis* , *Gelidium corneum* , *Gelidium spinolosum*, *Gigartina acicularis, Gracilaria multipartita*, *Osmundea pinnatifida*, and *Plocamium cartilagineum (*Rhodophyceae), *Ulva linza* and *Ulva* linza (Chlorophyceae), as well as *Laminaria ochroleuca* , *Cystoseira tamariscifolia, Bifurcaria bifurcata*, *Fucus spiralis* , and *Cystoseira humilis* (Phaeophycea).

Oumaskour et al. (2013) further investigated the influence of solvent polarity on the antibacterial effects of 23 algae including *H. incurva* . Six extracts were used (Hexane, methanol, acetone, dichloromethane‐methanol, chloroform, and water) and were tested against ten gram‐positive bacteria and two gram‐negative bacteria. In agreement with the findings of Farid and Etahiri (2009), the dichloromethane‐methanol extract showed strong activity against *Bacillus cereus* and moderate activity against *Staphylococcus aureus* and *Clostridium sporogenes*. The acetone extract exhibited high activity against *Staphylococcus aureus* ssp. *aureus*, while the methanol extract showed moderate activity against *Clostridium sporogenes*. However, the chloroform and water extracts were inactive. The antibacterial effectiveness varied according to the algal species, the solvent used, and the tested bacterial strains; notably, none of the screened algae exhibited activity against gram‐negative bacteria.

In contrast, Khelil‐Radji et al. (2017) revealed that the ethanolic extract of *H. incurva* was effective against both gram‐positive and gram‐negative strains, with pronounced inhibition zones reaching 22 mm against *Pseudomonas Aeruginosa* and 18 mm against *Staphylococcus aureus* and *Bacillus subtilis*. Moderate activity was also observed against *Escherichia coli* . Whereases, in agreement with the findings of Farid and Etahiri (2009) no activity was detected for the aqueous extract. Notably, in that study *H. incurva* was considerably more effective than *Halopteris scoparia* against *Staphylococcus aureus* and *Pseudomonas aeruginosa* , a difference that may be attributed to variations in their respective phytochemical compositions.

In another survey, the methanol extract of 44 seaweeds belonging to different taxonomic groups was screened (González del Val et al. 2001). *H. incurva* exhibited a significant inhibition zone against *Bacillus subtilis; however*, in contrast to the findings of Khelil‐Radji et al. (2017), no activity was detected against *Pseudomonas aeruginosa*. Among the screened Rhodophycea, *H. incurva* together with *Corallina elongata* showed the strongest antibacterial activity.

Overall, the presented evidence highlights *H. incurva* as a promising marine source of antibacterial agents. Variations in antibacterial activity appear to be influenced by solvent polarity, seasonal factors, and geographical origin, underscoring the need for bioactivity guided isolation and characterization studies to identify the compounds responsible for its antibacterial effects.

#### 4.1.2 Anti‐Mycobacterial Activity

*Mycobacterium tuberculosis* has developed wide drug resistance due to its ability to evade the mechanisms of action of several antimycobacterial agents currently used in tuberculosis treatment (Kapp et al. 2021). Therefore, there is an urgency to identify potent phytocompounds with favorable physicochemical properties. Allmendinger et al. (2010) screened the crude extract of 23 Rhodophyta, including *H. incurva*. To the best of our knowledge, this study represents the first and only report evaluating the antimycobacterial potential of *H. incurva* . The results showed that *H. incurva* extract, similar to most screened species, exhibited weak activity with a minimum inhibitory concentration. These findings suggest that although *H. incurva* displays weak antimycobacterial activity in crude extract, further investigations using different extraction solvents, fractionation approaches, and additional mycobacterial strains are warranted to more comprehensively assess its potential.

#### 4.1.3 Antifungal Activity

The methanol extract of *H. incurva* was examined against the yeast strains *Candida albicans* and *Saccharomyces cerevisiae* (González del Val et al. 2001). The results showed that, similar to all screened Rhodophyta, the *H. incurva* extract was inactive, with no inhibition observed against either yeast strain. Further investigations are therefore required to reassess the antifungal potential of *H. incurva* using different extraction solvents and other strains. The lack of information regarding the sampling period limits interpretation and should be considered.

In another survey, *H. incurva* demonstrated notable antifungal effect (Khelil‐Radji et al. 2017). The ethanolic and aqueous extracts significantly inhibited the growth of *Fusarium oxysporum* f. sp. *albedinis* and *Penicillium* sp., with the water extract showing higher effectiveness. This contrasts with the antibacterial assays, in which the aqueous extract was inactive against all tested bacterial strains, suggesting that metabolites present in this extract may be more effective against fungal pathogens than against bacteria. Consequently, further chemical characterization and bioactivity guided isolation of the active compounds are required. Notably, in both antifungal assays, *H. incurva* extracts displayed stronger activity than those of *Halopteris scoparia* . Otherwise, Bahammou et al. (2021) investigated the methanolic extracts of 17 species of seaweeds, including *H. incurva* against phytopathogenic fungi *Botrytis cinerea* and *Penicillium digitatum*, which infect fruits and vegetables. Antifungal activity of *H. incurva* was season‐dependent, with inhibition observed only in winter against *Penicillium digitatum* and in spring against *Botrytis cinerea*., with *H. incurva* ranking among the most potent species against *Botrytis cinerea*. Seasonal variation may modulate the chemical composition of *H. incurva* , potentially impacting its antifungal properties.

In another survey, the antifungal activity of the water extracts of 17 seaweeds and cyanobacteria, including *H. incurva* , was evaluated against *Podosphaera xanthii on zucchini* (Roberti et al. 2016). *H. incurva* demonstrated higher potency than the tested seaweeds in reducing the infected area by 96.8% and decreasing fungal sporulation by 30.2% relative to the inoculated control. Conventionally, powdery mildew is controlled using synthetic fungicides; however, their excessive and prolonged use has raised serious concerns regarding human and animal health, as well as environmental sustainability. In this context, the exploitation of natural products such as algal extracts represents a promising and eco‐friendly alternative for the management of phytopathogenic fungi.

#### 4.1.4 Antiparasitic Activities

##### 4.1.4.1 Leishmanicidal Activity

Lishmaniasis remains a major global health challenge in the 21st century, particularly in developing and underdeveloped regions. According to the World Health Organization, approximately one million new cases occur annually, with an estimated mortality exceeding 20,000 deaths per year (WHO 2023a). Leishmaniasis is classified as a neglected vector‐borne disease caused by protozoan parasites of the genus *Leishmania* (WHO 2023a). Current therapeutic options are limited and are often associated with high toxicity, increasing drug resistance, and elevated costs, highlighting the urgent need for safer and more effective antiparasitic agents. Allmendinger et al. (2010) investigated the leishmanicidal activity of 23 Rhodophyta species, including *H. incurva* against axenic amastigotes of *Leishmania donovani*. Their findings demonstrated that *H. incurva* exhibited the highest potential among all screened red algae (*Boergeseniella fruticulosa*, *Calliblepharis jubata* , *Ceramium virgatum* , *Chylocladia verticillata* , *Corallina officinalis* , *Claviclonium ovatum*, *Cystoclonium purpureum* , *Dumontia incrassata* , *Furcellaria lumbricalis*, *Gracilaria gracilis* , *Gelidium pulchellum* , *Gracilaria gracilis* , *Halurus equisetifolius* , *Jania rubens* , *Lomentaria articulata, Mastocarpus stellatus*, *Osmundea hybrida*, *Osmundea pinnatifida*, *Plocamium cartilagineum* , *Polyides rotundus* , *Porphyra linearis* , and *Porphyra leucosticte*). To the best of our knowledge, this study remains the first and only report describing the leishmanicidal activity of *H. incurva* . Therefore, further investigations are required to elucidate the molecular mechanisms underlying this promising bioactivity, alongside comprehensive chemical characterization to identify the compounds responsible for the observed effect.

##### 4.1.4.2 Trypanocidal Activity

Allmendinger and colleagues also attempted to unravel the trypanocidal activity of *H. incurva* against two protozoan *Trypanosoma brucei rhodesiense* and *Trypanosom Cruzi* (Allmendinger et al. 2010). *T. brucei rhodesiense* is the causative agent of Human African trypanosomiasis (sleeping sickness), an acute and often fatal disease endemic to 13 countries in eastern and southern Africa, accounting for approximately 8% of reported cases (WHO 2024b). In contrast, *T. cruzi* causes Chagas disease (American trypanosomiasis), a chronic systemic infection affecting 6–7 million people worldwide, primarily in Latin America, with up to one‐third of patients developing cardiac complications and around 10% presenting digestive or neurological disorder (WHO 2024a). Both diseases are classified as neglected tropical diseases. The results provided by Allmendinger et al. (2010) demonstrated that *H. incurva* exhibited significant trypanocidal activity against *Trypanosoma brucei rhodesiense*, ranking among the six most potent screened samples out of 23 Rhodophyta species screnned. However, against *Trypanosom. Cruzi*. In contrast, all algal extracts, including *H. incurva* , showed weak or no inhibitory activity against *T. cruzi* , even at the highest concentrations tested.

Thus, this activity has so far been investigated only once and remains the sole report revealing the trypanocidal potential of *H. incurva* . Molecular level studies are therefore required to elucidate the underlying mechanisms of action and parasite compound interactions responsible for the observed activity against *Trypanosoma brucei rhodesiense*. In addition, the use of alternative extraction solvents and fractions should be considered to reassess the apparent lack of activity against *T. cruzi* and to confirm this inactivity. To date, Allmendinger et al. (2010) remain the only study addressing these antiparasitic properties of *H. incurva* . Consequently, further investigations are warranted to fully explore its antiparasitic potential by screening additional clinically relevant protozoan strains, such as *Trypanosoma brucei gambiense*, another causative agent of Human African trypanosomiasis. Moreover, comprehensive chemical characterization and mechanistic studies are essential, particularly considering the potential influence of environmental, seasonal, and extraction‐related factors on the chemical profile and bioactivity of *H. incurva* .

##### 4.1.4.3 Molluscicidal Activity

Schistosomiasis is an acute and chronic parasitic disease that represents a major public health burden, particularly in South America, Asia, Africa, and the Caribbean (OMS 2022). It is caused by trematode worms of the genus Schistosoma, with human infection typically occurring through skin contact with freshwater contaminated by cercariae released from infected gastropod hosts, mainly snails of the genus *Biomphalaria* (OMS 2022). The larvae of this species develop in various snail species of the *Biomphalaria* genus. Consequently, disease control strategies often rely on reducing snail populations in transmission sites through the use of molluscicides (Patel et al. 2008). In this context, Patel et al. (2008) screened the aqueous fractions of methanolic extracts from 60 seaweed species, including *H. incurva* for molluscoid activity against *Biomphalaria glabrata* , a major intermediate host of Schistosoma (Patel et al. 2008). The results showed that most screened species were inactive; however, extracts from *Fucus vesiculosus* , *Fucus serratus* , *Dictyota dichotoma* , *Halidrys siliquosa*, *Pelvetia canaliculata* , and *H. incurva* killed all the test snails with LC_50_ = 100%. Notably, the molluscicidal activity of *H. incurva* was lost following further extract processing, including polyvinylpolypyrrolidone (PVPP) treatment and fractionation into low‐ and high‐molecular‐weight fractions by dialysis. In contrast, other active species retained activity after fractionation. These findings suggest that extraction and fractionation procedures markedly influence the molluscicidal activity of *H. incurva* , likely due to alterations in its chemical composition.

##### 4.1.4.4 Antiplasmodial Activity

Malaria is a neglected tropical disease caused by *Plasmodium* spp. and transmitted to humans through the bites of infected female *Anopheles* mosquitoes. The infection involves an initial asymptomatic liver stage followed by a symptomatic blood stage (Allmendinger et al. 2010; WHO 2023b). Infection in the human host begins when an infected Anopheles mosquito releases sporozoites into the bloodstream during a blood meal. These sporozoites migrate to the liver, where they multiply as liver‐stage (exo‐erythrocytic) forms over approximately 1 week, corresponding to an asymptomatic incubation period. The parasites then re‐enter the bloodstream and invade erythrocytes, initiating the symptomatic blood stage of infection (Allmendinger et al. 2010; WHO 2023b). Malaria predominantly affects tropical and subtropical regions, with the majority of cases and malaria related deaths occurring in Africa, where *Plasmodium falciparum* (WHO 2023b).

Spavieri et al. (2013) evaluated the chemotherapeutic and prophylactic potential of 23 algal extracts (CHCl_3_: MeOH [3:1 and 1:1]). Extracts were screened against both blood stage (BS) and the liver stage (LS) of *Plasmodium*, as well as against three key enzymes of the *P. falciparum* type II fatty acid synthase (FAS‐II system), β‐hydroxyacyl‐ACP dehydratase (PfFabZ), and β‐ketoacyl‐ACP reductase (PfFabG), and Enoyl‐ACP reductase (PfFabI). *H. incurva* displayed a weak activity for the blood stage compared with *Corallina officinalis L*., *Ceramium virgatum*, and *Osmundea pinnatifida* which exhibited strong activity (8.6, 13.6, and 14.5 μg/mL, respectively). In contrast, *H. incurva* exhibited notable liver stage inhibition, ranking second among all screened species. *H. incurva* exhibited strong inhibition of PfFabI, ranking third among the 23 screened seaweeds and showing greater potency than all other Rhodophyta species evaluated. Furthermore, *H. incurva* exhibited the strongest activity against PfFabZ, surpassing all tested red seaweeds and matching the activity of *Claviclonium ovatum*, while most other samples were inactive. Most of the tested samples showed no activity. Additionally, *H. incura* demonstrated notable inhibition against PfFabG. Notably, *H. incurva* also showed measurable inhibitory activity against PfFabG, further supporting its multi target antiplasmodial potential. Overall, only four of the screened species exhibited significant antiplasmodial activity, with *H. incurva* ranking second among the Rhodophyta and fourth among all 23 evaluated seaweeds. Five species showed weak activity (IC_50_ > 50 μg/mL), whereas the remaining 14 species were inactive.

**FIGURE 4:**
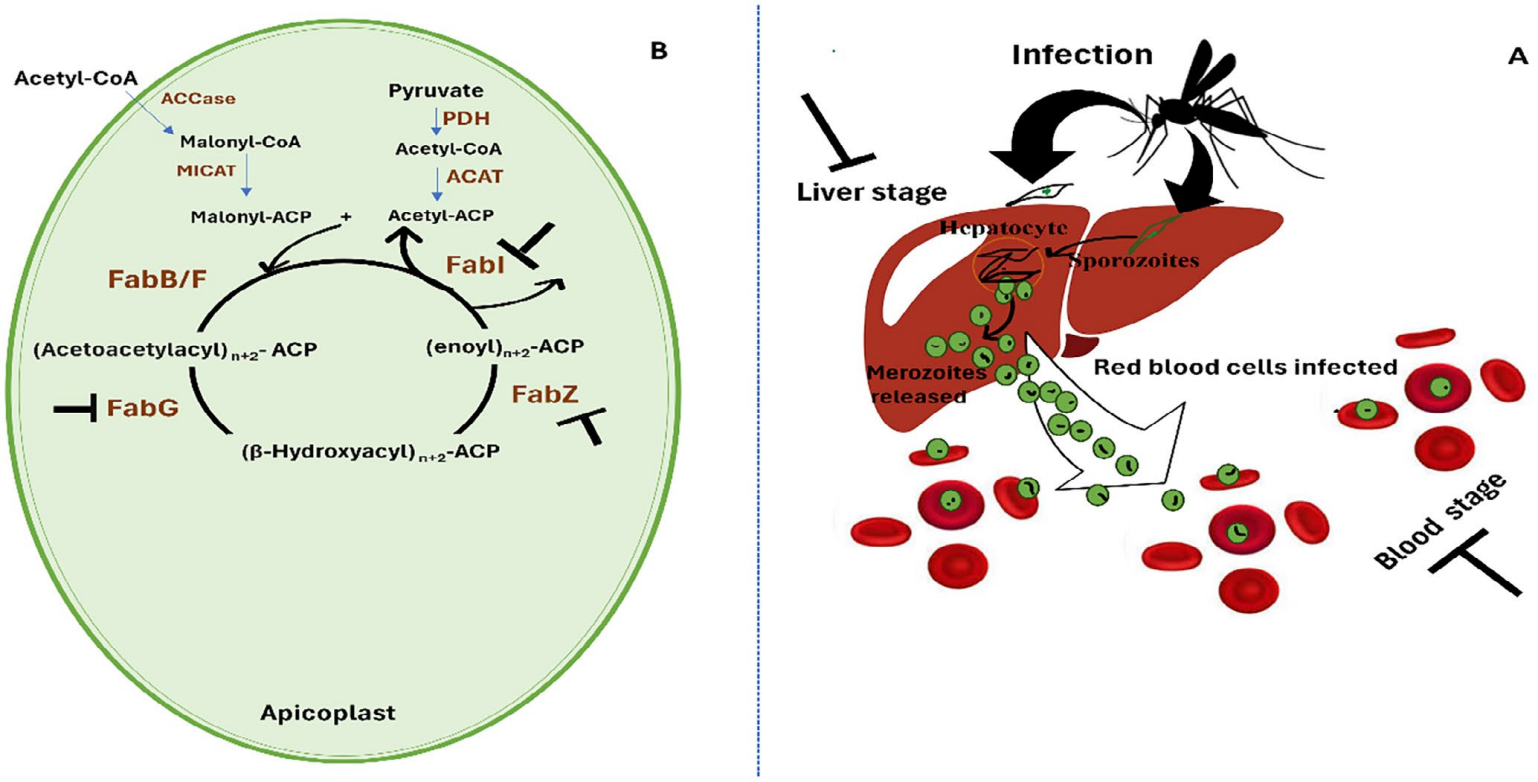
Antiplasmodial effect of *H. incurva* . (A) Inhibition of liver and blood stages of *Plasmodium* infection. After being injected by a mosquito, the released sporozoites first invade the liver, where they replicate and mature into merozoites within hepatocytes. Their replication and development lead to the rupture of hepatocytes, releasing the merozoites into the bloodstream. Once in the bloodstream, merozoites infect red blood cells, where they undergo further replication, producing daughter merozoites. This cycle repeats, resulting in the continuous infection of red blood cells. (B) Diagramatic representation of the biosynthesis of fatty acids via the FASII system in the apicoplast of *Plasmodium falciparum* and the inhibitory activity of *H. incurva* toward their key enzymes: Beta‐ketoacyl‐ACP reductase (FabG), enoyl‐ACP reductase (FabI), beta‐hydroxyacyl‐ACP dehydratase (PfFabZ), implicated in the elongation process of biosynthesis of fatty acid via FASII. In brown, the key enzymes are involved in fatty acid biosynthesis. Acetyl‐CoA carboxylase (ACCase), malonyl‐CoA‐ACP transacylase (MCAT), pyruvate dehydrogenase (PDH), and cholesterol acyltransferase (ACAT), PEP (phosphoenolpyruvate), and PK (pyruvate kinase) as intermediates in metabolism. The *n* + 2 displayed the condensation of two carbons into the nascent fatty acid chain.

These findings strongly support *H. incurva* as a promising marine source of multi‐target antiplasmodial agents. Such a broad inhibitory profile highlights its capacity to interfere with multiple nodes of the *Plasmodium* (FAS‐II) pathway. This survey appears the first and the only to investigate the antiplasmodial activity of *H. incurva*. Consequently, further investigations are warranted to elucidate its chemical composition, identify the bioactive compounds responsible for this activity, and clarify the underlying molecular mechanisms. Future studies should also explore additional targets within and upstream of the FAS‐II pathway, including FabB/F is which catalyzes the initial condensation step of fatty acid elongation, and FabH, which enables bypass of this step during early elongation cycles (Shears et al. 2015; Vaughan et al. 2009). Moreover, acyl‐carrier protein synthase (ACPS), a bifunctional enzyme essential for FAS‐II progression (Shears et al. 2015), and plastidic phosphate transporters (pPTs), which mediate phosphoenolpyruvate import into the apicoplast, represent attractive complementary targets (Shears et al. 2015). Inhibiting these processes could disrupt acetyl‐ACP and malonyl‐ACP formation, thereby impairing the initiation and continuation of FAS‐II‐dependent lipid biosynthesis (Shears et al. 2015; Todorinova et al. 2023). The ihnibitory activity of *H. incurva* against the liver and blood stages of *Plasmodium* infection is illustrated in Figure 4A, while Figure 4B depicts the fatty acid biosynthesis pathway via the FAS‐II system, highlighting the elongation enzymes and their inhibition by *H. incurva* .

### 4.2 Antioxidant Activity

Oxidative stress is defined as an imbalance between the excessive production of reactive species (pro‐oxidant stress) and their neutralization by antioxidant defense system (Hussain et al. 2016). Under physiological conditions, the human body is equipped with a robust network of enzymatic and non‐enzymatic antioxidants that regulate redox homeostasis. However, during infections or pathological states, oxidative stress may become persistent and inadequately counterbalanced, overwhelming antioxidant defenses (Ramos‐González et al. 2024). Consequently, oxidative stress is widely recognized as a key contributor in the onset and progression of numerous disorders (Hussain et al. 2016; Reddy 2023). In recent years, seaweeds have been extensively investigated for their potent antioxidant properties. In this regard, Nmila and Rchid (2018) evaluated the solvent polarity effect (crude, isopropanol, and chloroform), on the antioxidant potential of *H. incurva* . The DPPH assay was assessed by monitoring the percentage of remaining DPPH radical across different concentration ranges. The crude extract concentrations ranged from 0.025 to 1 mg/mL, showing DPPH% remaining of 83.70%, and 7.41%, respectively, as showing at 0.3 mg/mL (20%). The isopropanol extract concentrations ranged from 0.2 to 1.8 mg/mL, showing the remaining DPPH% of 85.52% and 35.34%, respectively. The chloroform extract concentrations ranged from 0.025 to 0.3 mg/mL, showing remaining DPPH% of 89.49% and 3.92%, respectively. As we note for the crude extract, at 0.3 mg/mL showed 20%. All extracts followed similar time‐ and concentration‐dependent kinetics, the DPPH% significantly decreasing when the concentration of the extract increased. Notably, the chloroform extract displayed the highest activity at the lowest concentrations. However, the results shown no significant difference between the obtained EC_50_ values of both crude and chloroform extracts, as well as both outperforming the reference (δ‐tocopherol), whereas the isopropanol extract was less active. These findings demonstrate that solvent polarity significantly influences the antioxidant potential of *H. incurva* , highlighting its strong free‐radical‐scavenging capacity and the need for further chemical characterization to identify the compounds responsible for this activity.

In another study the methanol and hydroalcoholic extracts of *H. incurva* alongside ten other seaweeds were examined (Güenaga Unzetabarrenechea 2011). The results ranked *H. incurva* among the most active species. In the DPPH assay both the methanol and hydroalcoholic extracts exhibited strong radical scavenging activity, with only slight difference between them. Moreover, in the FRAP assay both extracts exhibited the highest free radical scavenging activity among the tested seaweeds (*Cystoseira abies‐marina*, *Caulerpa racemosa* , *Hypnea spinella* , *Pterocladiella capillacea*, *Grateloupia dichotoma* , *Laurencia majuscula*, *Fucus spiralis* , *Ulva rígida*, and *Codium intertextum* ), with the hydroalcoholic extract displaying nearly double the activity of the methanolic extract. These findings indicate that solvent polarity significantly influences the antioxidant potential of the extracts. The authors attributed the pronounced activity of *H. incurva* to its high phenolic content. Vega et al. (2020) investigated the potential of both the methanolic (MeOH) and hydroalcoholic (EtOH 50%) extracts prepared from both wild‐collected and cultivated samples. In contrast to the findings of Nmila and Rchid (2018), in the DPPH assay the MeOH extract shown slightly more active than EtOH 50% extract, with no significant differences observed between collected and cultivated samples. However, marked differences were observed in the reducing power assay, which depended on both solvent and sampling procedure. The EtOH 50% exhibited higher activity than MeOH extract, and for both solvents, wild‐collected samples showed significantly greater activity than cultivated ones. Moreover, *H. incurva* demonstrated notably higher reducing power compared to the nine other screened seaweeds (*Treptacantha abies‐marina, Caulerpa racemosa*, *Dermocorynus dichotomus, Codium intertextum*, *Ulva rigida* , *Hypnea spinella, Pterocladiella capillacea, Laurencia dendroidea*, and *Fucus spiralis* ). A study carried out by Alvarez‐Gómez et al. (2016) investigated the influence of solvent on the antioxidant activity of *H. incurva* by examining four prepared extracts (H_2_O, 20% MeOH, 50% EtOH, and 100% EtOH). The results shown that no significant differences among the extracts in the β‐carotene assay. Similarly, in the ABTS assay, no significant differences were observed among the H_2_O, 20% MeOH, and 50% EtOH extracts, while the 100% EtOH extract exhibited slightly lower activity. In contrast, the DPPH assay revealed the highest antioxidant activity in the 20% MeOH, 50% EtOH, and 100% EtOH extracts, which showed comparable values, whereas the aqueous extract displayed the lowest activity. Notably, *H. incurva* exhibited higher activities across all three assays compared to *Gelidium corneum* , *Gelidium pusillum* , and *Ulva rotundata*.

In a subsequent study, the same authors evaluated the influence of ultraviolet radiation (UVR) on the antioxidant activity of the 20% MeOH extract using the ABTS assay (Álvarez‐Gómez et al. 2019). In initial time a similar anti‐ABTS activity of 12 μmol TE g^−1^ DW was found, which increased to 20 μmol TE g^−1^ DW after 4 days under PAB conditions. Although a slight decrease was observed between days 8 and 14, the activity remained elevated (18 μmol TE g^−1^ DW). Conversely, under PAR conditions, antioxidant activity decreased to 10 μmol TE g^−1^ DW after 4 days, returned to baseline by day 8, and remained stable thereafter. Overall, these findings indicate that solvent choice, together with UVR exposure, not only influences the chemical profile but also modulates the antioxidant potential of *H. incurva* in a time‐dependent manner. Importantly, the antioxidant response under both radiation regimes followed trends similar to those observed for total phenolic content suggesting that the observed variations may be largely attributed to changes in phenolic levels.

In another survey, the methanolic extracts of *H. incurva* and nine other Rhodophyta species harvested during August were investigated (Bouhlal et al. 2013). In contrast to the reported result of Vega et al. (2020), the methanolic extract of *H. incurva* was inactive in the DPPH assay. Indeed, the period of sampling may explain this difference. Whilst in the β‐carotene test, *H. incurva* ranked as the third strongest species compared to *Boergeseniella thuyoides, Ceramium rubrum, Gelidium spinulosum*, and *Hypnea musciformis*, as well as more effective than *Plocamium cartilagineum, Asparagopsis armata*, and *Sphaerococcus coronopifolius* which was inactive. Additionally, the aqueous extract of *H. incurva* showed an average scavenging activity of hydroxyl radical (%) using a deoxyribose test.

In another sense, the seasonal impact on the antioxidant activity of the hydroalcoholic extracts of *H. incurva* harvested in May (HIE/m) and November (HIE/n) was investigated (Vasarri et al. 2020). In the FRAP assay, the HIE/m extract exhibited markedly strong potential, approximately fivefold more than that HIE/n sample. Whereas, in the DPPH assay, the potential of HIE/m was nearly twofold lower than that of HIE/n. Notably, HIE/m contains TPC levels nearly twofold more than HIE/n, suggesting that antioxidant power may be attributed to its TPC levels. These results indicate that the antioxidant effectiveness of *H. incurva* is strongly influenced by seasonal variation and is assay‐dependent.

Furthermore, the antioxidant capacity of enzymatic protein hydrolysates (EPHs) derived from *H. incurva* was evaluated (Terriente Palacios et al. 2022). In both ABTS and DPPH assays, the EPHs exhibited significant antioxidant activity, highlighting proteins and their derived peptides as additional contributors to the overall antioxidant potential of *H. incurva*. In addition, the acetone/MeOH (1:1, 2 L) extract of *H. incurva* evaluated in the ABTS assay exhibited a significant percentage of inhibition at 400 μg/mL (Zbakh et al. 2014).

The influence of physicochemical parameters, particularly temperature, on the bioactivity of *H. incurva* extract was also studied (Plaza et al. 2010). In the ABTS assay, the subcritical water extraction obtained at 200°C showed stronger activity than that obtained at 100°C. A similar trend was observed in the ORAC_FL_ assay, in which *H. incurva* displayed the strongest activity among all screened algae. In contrast, the superoxide radical scavenging assay revealed higher activity for the extract obtained at 100°C compared to that extracted at 200°C. The enhanced ABTS and ORAC_FL_ activities of the extract produced at 200°C may be attributed to its higher total phenolic and carbohydrate contents, which increased at elevated extraction temperatures. Conversely, the superior superoxide radical scavenging capacity observed for the 100°C extract may be correlated with its higher amino acid content.

Considering the reported findings, extracts of *H. incurva* exhibit significant antioxidant potential across multiple assays, encompassing radical‐scavenging activity, reducing power, and total antioxidant capacity. However, their effectiveness appears to be influenced by several factors, including extraction solvent, methodological and procedural conditions, seasonal variation, and temperature. Therefore, further investigations should focus on comprehensive chemical characterization of these extracts to elucidate their phytochemical composition, identify the major contributing compounds, and systematically evaluate the antioxidant activity of individual constituents. In addition, chelating activity has not yet been investigated and represents an important avenue for future research.

### 4.3 Anti‐Inflammatory and Immunomodulatory Properties

Rhodophyta have been increasingly recognized for their potential in inflammation management (Balkrishna et al. 2025), and enzymatic inhibition particularly targeting elastase and phospholipase A_2_ (PLA_2_) is considered an effective anti‐inflammatory strategy. In this backdrop, Oumaskour and collaborators evaluated the anti‐inflammatory potential of dichloromethane/methanol (50:50) extracts of 23 Rhodophyta including *H. incurva* against elastase and PLA_2_ (Oumaskour et al. 2013). The results indicated that *H. incurva* exhibited weak activity against PLA_2_ compared to other species such as *Gelidium sesquipedale* , *Chondrus crispus* , and *Asparagopsis armata* , which showed an inhibition of 100%. Likewise, *H. incurva* displayed little to no elastase inhibition relative to *Chondrus crispus* , *Corallina elongata* , *Laurencia pinnatifida*, and *Gelidium sesquipedale* , which exhibited inhibition levels exceeding 95%. The limited activity observed for *H. incurva* may be related to the extraction solvent employed, particularly as other extracts prepared from *H. incurva* showed strong and multitarget antibacterial activity in the same study. This highlights the need for alternative extraction strategies, comprehensive chemical characterization, and bioactivity‐guided screening of isolated compounds against these and other key inflammatory enzymes.

**FIGURE 5:**
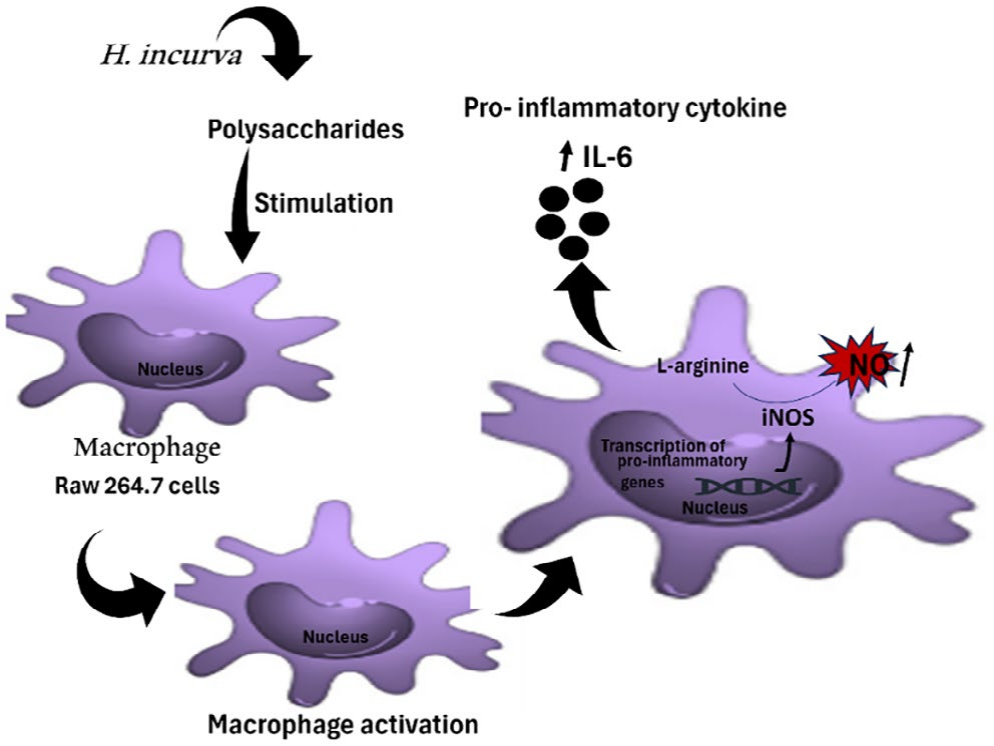
Immunomodulatory properties of *H. incurva* polysaccharides on macrophage (RAW 246.7), polysaccharides (contain cytokine inducers) stimulate macrophage, leading to activation and enhancing the sur‐production of pro‐inflammatory cytokines such as interleukin 6 (IL‐6). Also, the inducible form of nitric oxide synthase (iNOS) is responsible for NO production in macrophage, where the substrate is arginine.

In another investigation, the authors evaluated the immunomodulatory potential of the polysaccharides of *H. incurva* collected during spring period using RAW 264.7 macrophages (Abdala Díaz et al. 2011). The study assessed the macrophages proliferation and cytokine release, including tumor necrosis factor α (TNF‐α), nitric oxide (NO), and interleukin 6 (IL‐6). The findings revealed that NO and IL‐6 activation increased linearly with the increase of the *H. incurva* polysaccharides concentration, with levels approximately tenfold higher than those induced by *Hypnea spinella* polysaccharides. Nevertheless, IL‐6 production reached a plateau at concentrations above 50 μg/mL, while TNF‐α production and macrophage proliferation were not affected. These findings suggest that *H. incurva* polysaccharides act as cytokine IL‐6 and NO inducers, suggesting immunomodulatory properties potentially exerting either pro‐ or anti‐inflammatory effects depending on the biological context. However, further chemical characterization is needed to unravel the molecules responsible for these properties. The immunomodulatory effects of *H. incurva* polysaccharides on macrophage activation are illustrated in Figure 5.

The in vivo study conducted on zebrafish ( *Danio rerio* ) demonstrated that dietary supplementation with *H. incurva* exerted a pronounced positive effect on both immune and antioxidant responses (Hoseinifar et al. 2022). Fish fed diets containing *H. incurva* (0.25%–1%) exhibited significant increases in key innate immune parameters, including lysozyme activity, total immunoglobulin (Ig), and total protein levels in both whole‐body extract and skin mucus. Notably, the most pronounced enhancement of these immune parameters was consistently observed at the 0.5% inclusion level. In parallel, *H. incurva* supplementation significantly improved antioxidant capacity, as evidenced by increased activities of superoxide dismutase (SOD), catalase (CAT), and glutathione peroxidase (GPx), along with a reduction in malondialdehyde (MDA) levels. At the molecular level, dietary *H. incurva* also induced the upregulation of immune‐related genes (IL‐1β, TNF‐α, IFN‐γ) and antioxidant‐related genes (SOD and GPx), further confirming its immunostimulatory and antioxidant potential. Collectively, these findings indicate that dietary *H. incurva* , particularly at moderate inclusion levels, can significantly enhance innate immunity, antioxidant defense, and immune‐related gene expression in zebrafish.

### 4.4 Anti‐Amyloidogenic and Anti‐Tumoral Activities

Amyloid aggregation is regarded as the triggering event that leads to clinical symptoms of amyloidosis, ultimately resulting in neurotoxicity. Exploring novel strategies in the prevention of neurodegeneration, as well as seeking effective anti‐amyloidogenic agents remains a crucially needed and primordial way for amyloid research. In this regard, blocking amyloid aggregates formation by directly inhibiting the self‐assembly process has been proven effective.

Indeed, hen egg white lysozyme (HEWL) is widely employed as a model protein in amyloid aggregation studies to investigate the morphological features, kinetics, and mechanisms underlying amyloid fibrillation. The anti‐amyloidogenic potential of hydroalcoholic extracts of *H. incurva* harvested in November (HIE/n) and May (HIE/m) was evaluated by examining their effects on HEWL amyloid aggregation (Vasarri et al. 2020). The ThT fluorescence intensity of HEWL–HIE/m and HEWL–HIE/n aggregates decreased by approximately three‐fold and seven‐fold, respectively, compared to HEWL aggregated in the absence of HIEs. Interestingly, morphological analysis revealed that HEWL–HIE/m aggregates were noticeably shorter than those formed in the presence of HIE/n. This difference strongly reflects the impact of seasonality, which influences the chemical composition of *H. incurva* and, consequently, its biological activity. These findings demonstrate that the two HIE samples qualitatively and differentially modulate the amyloid aggregation process. Notably, amyloid fibrillation was markedly slowed in the presence of HIEs, making aggregate formation difficult even over extended incubation periods, with the aggregation kinetics reaching a plateau around day 10.

FM analysis was further employed to examine the morphology of HEWL‐aggregated species. After 10 days of incubation, HEWL alone led to the formation of well‐defined, mature fibrils characterized by long, unbranched morphologies. In contrast, samples incubated with HIEs exhibited a reduced fibrillar load, with the presence of mixed populations consisting of shorter fibrillar assemblies together with oligomeric and/or amorphous structures. These observations suggest that seasonal variations in the chemical composition of *H. incurva* play a decisive role in modulating the effects of HIEs on amyloid fibrillation. The authors also evaluated the cytotoxic and antiangiogenic activities of these extracts, using the MTT test on SH‐SY5Y human neuroblastoma cells. The results demonstrated that HIEs alone were safe for cells within the tested concentration ranges (0.11 μg/mL for HIE/m and 1 μg/mL for HIE/n). Moreover, HEWL amorphous fibrils formed in the presence of HIE/m were markedly less toxic than those formed with HIE/n or HEWL alone. Notably, the MTT assay revealed a significant recovery of cell viability of approximately 20% ± 2% in HEWL‐HIE/m‐treated cells compared to cells treated with HEWL alone. In another study, the antiproliferative activity of dichloromethane‐methanolic extract of *H. incurva* was investigated on the canine C2 cell line, using the Alamar Blue (AB) tests analysis (Amminikutty 2021).

The result highlighted that *H. incurva* extract exhibited weak antiproliferative activity compared to *Gracilaria cervicornis* and *Plocamium cartilagenium*. In the same interest, the antiproliferative activity of 23 Rhodophyta species, including *H. incurva* , was evaluated (Allmendinger et al. 2010). *H. incurva* was found to be non‐toxic to L6 cells, similar to most of the other species.

Nevertheless, in another study the acetone/MeOH extract of *H. incurva* presented a significant antiproliferative effect against HT29 colon cancer cells (Zbakh et al. 2014). Cells were treated with a range of extract concentrations ranging from 12.5 to 200 μg/mL for 48 and 72 h, revealing a clear dose‐ and time‐dependent effect on cell growth, with increased cytotoxicity at higher concentrations and longer exposure times. Collectively, these findings indicate that *H. incurva* extracts exhibit weak cytotoxicity toward non‐tumoral or non‐cancerous cell models (e.g., canine C2 and L6 cells), while showing more pronounced antiproliferative effects against mammalian cancer cell lines, depending on the extraction solvent and experimental conditions. These observations highlight the need for detailed chemical characterization of *H. incurva* extracts to more understanding its molecular intervention, as well as emphasize the importance of further studies using different extraction solvents to confirm and better understand the previously reported weak activity of this species.

### 4.5 Neuroprotective Activity

To date, only one study has investigated indirectly the neuroprotective potential of *H. incurva* , namely the work of Kilic et al. (2021), which evaluated its inhibitory activity against acetylcholinesterase (AChE) and butyrylcholinesterase (BChE). These enzymes are strongly implicated in the pathogenesis of neurodegenerative diseases (Moss and Perez 2021) and have also recently attracted attention as potential targets for the development of novel insecticides, which constituted the primary focus of the authors' investigation (Kilic et al. 2021). Among the 47 macroalgae screened, *H. incurva* was identified as one of the active species against both enzymes. Specifically, only 10 out of 47 species exhibited measurable AChE inhibition, with *H. incurva* ranking fourth in potency. For BChE inhibition, it ranked 21st among the 30 macroalgae that showed inhibitory activity. Based on these findings, further investigations using other extracts are required to comprehensively assess the potential of this species, as well a detailed chemical characterization of the ethanolic extract is warranted, followed by complementary in vitro and *in silico* studies to elucidate the compounds responsible for the observed activity. The observed inhibitory activity of *H. incurva* together with its reported chemical profile rich in GABA, Taurine, and SUM (Terriente Palacios et al. 2022), supports its neuromodulatory and cytoprotective properties. Notably, MAAs and carbohydrate from red algae have been shown to exhibit strong neuroprotective and immunomodulatory activities (Ferreira et al. 2026). In addition, further investigations using extracts obtained with different solvents are required to comprehensively assess the neuroprotective potential of this species.

## 5 Conclusion and Perspectives

*Halopithys incurva* (Hudson) Batters, 1902 is an underexplored red macroalga characterized by a rich and multifaceted biochemical composition, encompassing both primary nutritional constituents and bioactive secondary metabolites. Available studies report the presence of several bioactive compounds, including bromophenols, mycosporine‐like amino acids, and isoflavones. In addition, this species contains appreciable levels of proteins, carbohydrates, and diverse pigments, such as chlorophyll a, carotenoids, and phycobiliproteins, pigments characteristic of Rhodophyta. The presence of GABA and taurine has also been reported. Together, these components contribute to the nutritional value and bioactivity of *H. incurva* . Lipidomic investigations further indicate that *H. incurva* is distinguished by a relatively high proportion of polyunsaturated fatty acids (PUFAs), particularly α‐linolenic acid and eicosapentaenoic acid compared with several other screened marine macroalgae. In accordance with this favorable fatty acid composition, reported nutritional lipid indices, including low atherogenicity (AI) and thrombogenicity (TI) indices, suggest a balanced lipid profile from a cardiovascular nutrition perspective. Together, its chemical composition profile underscores the biochemical and nutritional interest of *H. incurva* in comparative macroalgal studies. Beyond compositional aspects, extracts and polysaccharide fractions of *H. incurva* have been shown to exhibit a broad range of biological activities. While most reported effects are supported by in vitro evidence, limited in vivo investigations indicate that dietary supplementation with *H. incurva* can enhance systemic and mucosal immune responses and improve antioxidant status, accompanied by modulation of immune‐ and oxidative stress–related gene expression. These findings provide preliminary organism‐level evidence of immunomodulatory and antioxidant effects, although the underlying mechanisms remain incompletely understood. Despite these promising biochemical and biological attributes, *H. incurva* has no documented history of traditional or modern human consumption. This absence of ethnobotanical or dietary use may be partly related to its geographical distribution, which is largely confined to specific coastal regions and often characterized by limited accessibility or lower historical integration into traditional food systems. In addition, comprehensive toxicological, nutritional, and food safety assessments are currently lacking, precluding any conclusions regarding its suitability for human consumption. Overall, the available evidence positions *H. incurva* as a valuable marine resource for bioprospecting, comparative nutritional assessment, and fundamental research rather than immediate food or therapeutic use. Future investigations should prioritize integrated chemical characterization, elucidation of structure–activity relationships, expanded in vivo validation, and rigorous toxicological and safety evaluations to clarify the biological relevance, practical potential, and regulatory feasibility of this species.

## Author Contributions

**Learn‐Han Lee:** writing – review and editing, methodology, investigation, conceptualization. **Gokhan Zengin:** data curation, supervision, visualization, writing – review and editing. **Youssra Aalilou:** writing – original draft, conceptualization, methodology, data curation. **Kaoutar Benrahou:** methodology and data curation. **Issam Ameziane El Hassani:** writing – review and editing, methodology, software. **Abdelhakim Bouyahya:** conceptualization, methodology, supervision, project administration, writing – review and editing. **Waleed Al Abdulmonem:** funding acquisition, investigation, writing – review and editing, methodology, validation. **My El Abbes Faouzi:** conceptualization, project administration, supervision, writing – review and editing. **Mustapha Hassoun:** data curation, methodology, formal analysis. **Hanaa Moussa:** data curation, investigation, writing – review and editing.

## Funding

This work was supported by the Deanship of Graduate Studies and Scientific Research at Qassim University for financial support (QU‐APC‐2026).

## Conflicts of Interest

The authors declare no conflicts of interest.

## Data Availability

The data that support the findings of this study are available from the corresponding author upon reasonable request.
